# *Helicobacter pylori–*activated fibroblasts as a silent partner in gastric cancer development

**DOI:** 10.1007/s10555-023-10122-1

**Published:** 2023-07-17

**Authors:** Gracjana Krzysiek-Maczka, Tomasz Brzozowski, Agata Ptak-Belowska

**Affiliations:** Department of Physiology, the Faculty of Medicine, Jagiellonian University Medical College, 16 Grzegorzecka Street, 31-531 Kraków, Poland

**Keywords:** *Helicobacter pylori–*induced fibroblast activation, Cancer-associated fibroblasts, Cell signaling, Gastric cancer, Stemness, Cell reprogramming

## Abstract

The discovery of *Helicobacter pylori* (*Hp*) infection of gastric mucosa leading to active chronic gastritis, gastroduodenal ulcers, and MALT lymphoma laid the groundwork for understanding of the general relationship between chronic infection, inflammation, and cancer. Nevertheless, this sequence of events is still far from full understanding with new players and mediators being constantly identified. Originally, the *Hp* virulence factors affecting mainly gastric epithelium were proposed to contribute considerably to gastric inflammation, ulceration, and cancer. Furthermore, it has been shown that *Hp* possesses the ability to penetrate the mucus layer and directly interact with stroma components including fibroblasts and myofibroblasts. These cells, which are the source of biophysical and biochemical signals providing the proper balance between cell proliferation and differentiation within gastric epithelial stem cell compartment, when exposed to *Hp*, can convert into cancer-associated fibroblast (CAF) phenotype. The crosstalk between fibroblasts and myofibroblasts with gastric epithelial cells including stem/progenitor cell niche involves several pathways mediated by non-coding RNAs, Wnt, BMP, TGF-β, and Notch signaling ligands. The current review concentrates on the consequences of *Hp*-induced increase in gastric fibroblast and myofibroblast number, and their activation towards CAFs with the emphasis to the altered communication between mesenchymal and epithelial cell compartment, which may lead to inflammation, epithelial stem cell overproliferation, disturbed differentiation, and gradual gastric cancer development. Thus, *Hp*-activated fibroblasts may constitute the target for anti-cancer treatment and, importantly, for the pharmacotherapies diminishing their activation particularly at the early stages of *Hp* infection.

## Introduction

It has been demonstrated that the first-class carcinogen *Helicobacter pylori* (*Hp*) infection within gastric mucosa leads to active chronic gastritis, gastroduodenal ulcers, and finally gastric cancer (GC). The *Hp* basic and clinical research has opened the new avenues and provided evidence for microbiological background for not only gastrointestinal (GI) disorders, but also for other extragastric diseases related to chronic inflammation. Inflammation triggered by *Hp* stimulates the release of many mediators such as a variety of cytokines, chemokines, and growth factors together with the activation of cellular effectors like gastric stem cells resulting in GC development [1–3]. Despite extensive research, the pathomechanism of the *Hp*-induced inflammation remains not fully understood. Originally, the *Hp* virulence factors affecting mainly gastric epithelium were proposed to evoke these events [4, 5]. Nevertheless, it has been shown that *Hp* may penetrate the mucus layer reaching pits of gastric glands and subsequently directly interacts with stroma components including fibroblasts [6–15].

## Gastric mucosa in homeostasis and GC development

### Epithelial stem cell compartment

**Fig. 1:**
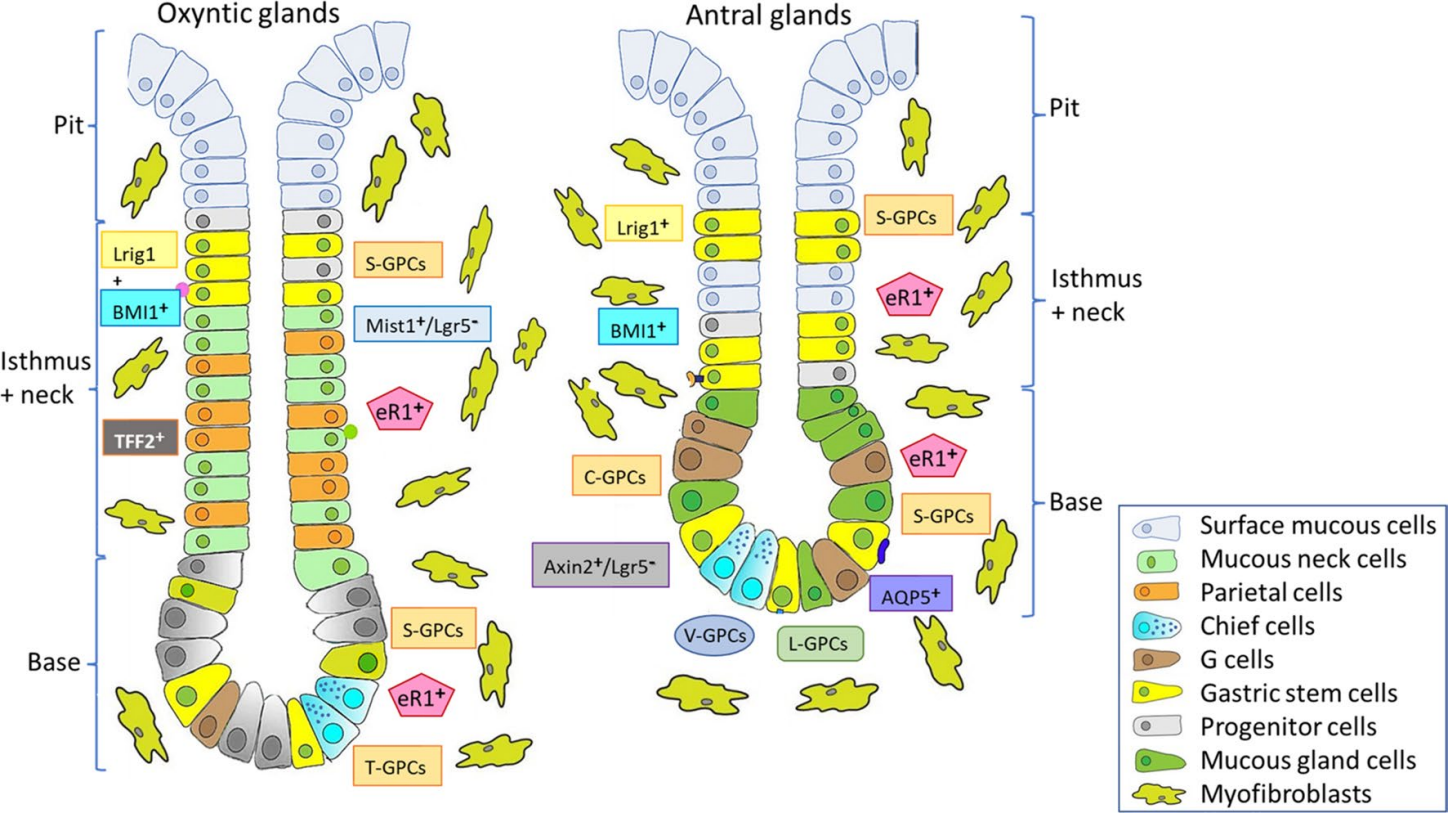
Schematic view on the stem and progenitor cell compartment within the oxyntic (corpus) and antral glands

The inner surface of the stomach is lined by a mucous membrane known as the gastric mucosa which consists of simple columnar epithelium, *lamina propria*, and *muscularis mucosae*. Due to the severe, acidic conditions in the stomach, gastric epithelial layer undergoes the process of continuous renewal within 3 days [16, 17] which depends upon constant cell proliferation. The gastric mucosa contains about 3 million pits, forming the entrance into the gastric glands consisting of the isthmus, the neck, and the base region. There are two types of gastric glands: a fundic type (mainly in the fundus/corpus zone) and an antral type (in the cardiac and the antral/pyloric zones) (Fig. 1). The combination of a pit and its associated glands is called a gastric unit [18, 19]. Fundic units contain five principal mature epithelial cell types, whereas the simpler antral units are made of three predominant types of mature epithelial cells. The isthmus and neck regions had been identified as the major site of proliferation [19, 20] containing gastric epithelial stem cells which replenish lost or damaged cells of gastric mucosa [21]. Originally, the isthmus was believed to exclusively harbor gastric stem cells [19, 22, 23]. Nevertheless, clonal tracing study in the human stomach showed multiple stem cells present within the single gastric unit, but each individual gland seems to be populated by the progeny of a single stem cell [18, 19]. It has been demonstrated that a single stem cell can also expand and colonize the entire unit in a process called monoclonal conversion [18, 19]. Qiao et al. [24] identified villin promoter–marked cells (V-GPCs) mainly located in the base of antral glands (Fig. 1), which represent a highly quiescent stem cell that only becomes apparent upon stimulation by interferon γ [19, 24] and able to differentiate into all types of antral gland cells [17]. LGR5-positive gastric stem cells (L-GPCs) were identified [19, 25, 26] and their presence in humans was demonstrated at the base of antral glands but not in fundic units (Fig. 1) [19, 25, 27]. The lineage tracing demonstrated these cells function as multipotent, self-renewing stem cells which are capable of building long-lived gastric organoids *in vitro* [19, 25]. In the same zone, a group of AXIN2^+^/LGR5^−^ stem cells that can give rise to LGR5^+^ cells were identified (Fig. 1) [28]. The high proliferation rate of these cells depended on the presence of Wnt agonist R-spondin (RSPO) [17, 29].

Another stem cell population characterized by expression of the gastrin/CCK2 receptor LGR5^neg or low^ (C-GPCs) has been identified in murine antral glands [27]. C-GPCs are localized slightly above typical L-GPCs (Fig. 1) [19] and do not overlap with them at the antral gastric units; however, when treated with progastrin, C-GPCs interconvert into typical antral L-GPC cells [17, 19, 27]. Additionally, aquaporin 5 (AQP5) expressing populations were identified to overlap with LGR5^+^ stem cells at the base of pyloric gland (Fig. 1), where they comprise the pyloric lineages expressing Muc5AC, gastric intrinsic factor, gastrin, and chromogranin A [17, 30]. It has been shown that gastric mucus neck cells, located below the isthmus, express trefoil factor family 2 (TFF2) protein (Fig. 1), while TFF2 mRNA transcripts are concentrated in cells above the neck region in normal corpus mucosa, suggesting that TFF2 transcription is a marker of gastric progenitor cells [31, 32]. SOX2^+^ stem and progenitor cells (S-GPCs) were discovered in the mice stomach [33]. These cells are scattered throughout the isthmus in both the fundic and antral units as well as in lower parts of the glands (Fig. 1) and they give rise to all mature gastric cell types [17, 19, 21, 33]. Within the corpus isthmus, MIST1^+^/LGR5^–^ cells (Fig. 1) were shown to be capable of giving rise to surface mucus cells, mucus neck cells, parietal cells, tuft cells, and enterochromaffin-like cells (ECL) [17, 34, 35]. According to Hayakawa et al. [34, 36], these quiescent stem cells may play a critical role in differentiation of corpus glands. *In vitro*, MIST1^+^ isthmus cells can form corpus organoids, whereas MIST1^+^ chief cells remain single [34]. Additionally, a subgroup of fully differentiated TROY^+^ chief cells (T-GPCs) is capable of regaining stem cell capacity. T-GPCs at the base of gastric corpus units (Fig. 1) are considered the reserve quiescent stem cells which can re-enter the cell cycle and act as multipotent stem cells giving rise to all gastric units following tissue damage due to the process of dedifferentiation or transdifferentiation [17, 19, 26]. Additionally, Matsuo et al. [37] have reported that corpus cells labeled by a Runx1 enhancer element (eR1) present in the isthmus and base of pyloric glands (Fig. 1) are involved in tissue regeneration and continuously give rise to mature cells that maintain gastric units [17, 37]. Choi et al. have shown that Lrig1-expressing isthmal cells (Fig. 1) can contribute to the regeneration of parietal cells following acute gastric injury [17, 38]. BMI1-expressing cells in the isthmus of gastric antrum and corpus (Fig. 1) were shown to provide progeny both towards luminal and basal sides, although it is not clear whether they are colocalized with other reported stem cells [17, 28, 39]. Summarizing, gastric stem cell populations include V-GPCs, L-GPCs, C-GPCs, S-GPCs, AXIN2+, eR1+, Bmi1+, Lrig1+, and AQP5+ stem cells in the antrum as well as Mist1+ and T-GPCs in the corpus. S-GPCs, eR1+, Lrig1+, and Bmi+ marked cells are present both in the antrum and corpus [17, 19].

### Cancer stem cells

Accumulating evidence revealed an intimate link between stem cells and GC formation [40]. Nowadays, cancer is thought to derive from a small subset of cancer stem cells (CSCs) [17, 19, 27, 33, 34, 36, 40, 41], which share features with true stem cells by possessing the capacity to self-renew in de-differentiated state, to generate heterogeneous types of differentiated progeny, and to give rise to the bulk tumor [42, 43]. This idea has also been proven for GC and numerous signaling pathways have been implicated in stem cell homeostasis, including Hedgehog, Wnt, Notch, various miRNAs, EGF receptor ligands, TGF-β/BMP, and NF-κB [19, 39, 44–46]. Currently, at least three different cellular origins of gastric CSCs have been suggested. Gastric CSCs are likely the result from dedifferentiation of metaplastic lineages including spasmolytic polypeptide expressing metaplasia (SPEM) and intestinal metaplasia (IM), particularly in the corpus [19, 47–49]. The second source of CSCs can be bone marrow–derived mesenchymal stem cells (BM-MSCs) which have been shown to be able to engraft into the gastric mucosa and lead to adenocarcinomas under the conditions of chronic inflammation [19, 22, 45, 50, 51]. Additionally, their differentiation pattern mainly depends upon the local microenvironment [17, 19, 22, 45, 52]. Thirdly, gastric CSCs may also be generated from normal adult gastric progenitor cells (GPCs)/stem cells [17–19, 45]. Xiao and Zhou [17] indicated long-lived stem cells as the ideal cellular target for the accumulation of mutations under the action of external environments such as continuous action of inflammatory mediators and stimulation by carcinogens [17]. It has also been shown that targeted deletion of reprogramming-related transcription factor Klf-4 or peroxisome proliferator–activated receptor delta (*PPARD*) overexpression in V-GPCs induced transformation, tumorigenesis in the antrum of mice, and GC development [17, 53, 54]. Studies in mice have documented that LGR5^+^ epithelial stem cell pool in the antrum was more susceptible to DNA damage than LGR5^–^ cells [17, 53]. Consequently, Cancer Genome Atlas (CGA) database analysis together with immunohistologic staining revealed that the intestinal adenocarcinomas of gastric antrum and gastroesophageal junction were accompanied by the expansion of L-GPCs [17, 55]. Additionally, SOX-2^+^ cells have been identified as the source of CSCs in GC [56]. Importantly, *Hp* infection has been shown to disturb homeostasis in the gastric stem cell niche, leading to malignant transformation. Siegal et al. visualized bacteria microcolonies in the neck region and base of gastric antral glands of both mice model and human specimens infected with *Hp*. He has showed that CagA^+^ *Hp* directly accelerated L-GPC-derived gland turnover at the early stage after infection [17, 19, 29]. The increased level of progastrin demonstrated in *Hp*-positive GC patients [57] has been shown to convert C-GPC cells into L-GPC cells, to increase the number of C-GPCs, and to promote gland fission and carcinogenesis in response to the chemical carcinogen *N*-methyl-*N*-nitrosourea (MNU). Pharmacological inhibition or genetic ablation of C-GPCs has been shown to attenuate progastrin-dependent stem cell expansion and carcinogenesis [17, 27]. Also, Lie et al. [58] have confirmed that gastric L-GPCs may act as the cancer-initiating cells contributing to malignant progression in the *Hp*-infected mouse model. The deletion of tumor suppressive SMAD4 and antiproliferative Pten L-GPCs led to the progression from microadenoma and macroscopic adenoma to invasive intestinal-type GC in the gastric antrum [17, 58]. Additionally, it has been found that another species from the *Helicobacter* family—*Helicobacter felis*—was shown to induce inflammation and to inhibit BMP signaling, leading to activation of L-GPCs as well as to metaplastic and dysplastic transformation of epithelial cell lineages [17, 59]. Leushacke et al. [60] defined LGR5^+^ chief L-GPC cells as a major source of GC. For GC in the corpus, MIST1^+^ isthmus stem cells can serve as an origin. In combination with *Helicobacter* species infection, deletion of *Cdh1* gene in MIST1^+^ isthmus stem cells resulted in diffuse-type GC, whereas intestinal-type adenocarcinoma was induced by ablation of Aps and Kras mutation in these cells [17, 34]. Wnt pathway activation in gastric stem cells has been associated with gastric carcinogenesis [17, 61, 62]. It has been shown that during *Helicobacter felis* infection, Mist1^+^ isthmus stem cells can be activated in part through Wnt 5a [35] and AQP5^+^ stem cells were identified as a source of Wnt-driven GC [17, 63]. Additionally, Zhao et al. [64] have identified coexpression of AQP5 with LGR5 in CSCs isolated from GC patients. Consequently, it has been found that during chronic inflammation induced by *Hp*, some chief cells arising from Lrig1^+^ populations [65] might contribute to the development of pseudopyloric metaplasia/spasmolytic polypeptide-expressing metaplasia (SPEM) [17, 66]. Therefore, it can be assumed that *Hp* infection may contribute to GC by influencing the imbalance between gastric epithelial turnover and stem cell differentiation.

### Fibroblasts and myofibroblasts

**Fibroblasts** are the principal active cells of connective tissue which support the structure and function of GI organs and maintain its barrier integrity. They are capable of producing, modifying, and organizing extracellular matrix (ECM) components [67, 68] together with its specialized dense, tension-resistant form known as the basement membrane (BM) [69, 70]. Both ECM and BM constitute the source of both the biophysical and biochemical cues necessary for cell attachment, polarity, and migration within and across tissue compartments [69, 71, 72]. Consequently, it has been shown that the anterior-posterior regionalization of epithelial cells in the GI tract is organized by mesenchymal cells [73, 74].

Under normal, physiologic conditions, epithelial cells are separated from fibroblasts in the adjacent connective tissue [69, 75] by the BM. Non-activated or quiescent fibroblasts showing minimal metabolic and transcriptional activity [69, 76] are dispersed within the stromal tissue compartment. Myofibroblasts are distinguished from fibroblasts by their enhanced contractile properties and the presence of α-smooth muscle actin (α-SMA). Within the GI tract, small amount of myofibroblasts reside mainly within subepithelial layer immediately beneath the epithelial BM and are known as pericryptal fibroblasts [77–79]. Fibroblasts and myofibroblasts orient themselves in the direction of matrix fibers, without direct association with the BM [69, 80]. Despite decreased overall activity, fibroblasts and myofibroblasts provide specific biophysical and biochemical signaling which influence the key processes fundamental for the proper tissue function [68]. The intestinal subepithelial myofibroblasts have been shown to contribute to the regional epithelial differences along the alimentary tract, for example, organizing endoderm into well-differentiated villi, containing enterocytes, goblet cells, and enteroendocrine cells [21, 78, 81–83]. Both fibroblasts and myofibroblasts release a cocktail of humoral factors including growth factors, cytokines, and chemokines responsible for the maintenance of the proper balance between proliferation and differentiation of other cell types including epithelial cells [12, 13]. Myofibroblasts have been proposed to support LGR5^+^ stem cell niches within gastric pits [21, 25, 77, 81–84], constituting important source of Wnt, BMP, or TGF-β signaling ligands engaged in maintaining epithelial stem cell niches and differentiation towards specialized progeny [21, 73, 81–84]. Additionally, fibroblasts are nowadays acknowledged as a “non-classical” branch of the innate immune system due to their role of key immune sentinels, which activate and modulate immune responses upon the detection of pathological stimuli. Concomitantly, fibroblasts have been shown to detect damage-associated molecular patterns and pathogen-associated molecular patterns, activating pro-inflammatory signaling pathways which play the role in leukocyte recruitment and activation [84]. It has been shown that under specific conditions, such as a wound repairing process, the mesenchymal cells in the peritoneal area migrate to the wound bed and contribute to proliferation and structural morphogenesis of intestinal epithelium *via* non-canonical Wnt signaling [73, 82, 85, 86]. During wounding, the mechanical damage as well as acute inflammation breaks down physical barrier between epithelial and stromal cells. The reactive stroma is characterized by the appearance of increased number of myofibroblasts in the area surrounding inflammatory region. Differentiation of fibroblasts towards myofibroblasts is defined as fibroblast-to-myofibroblast transition (FMyoT) and occurs due to the different kinds of stimuli with crucial role of auto- and paracrine TGF-β signaling in this process [68].

The physiological role of fibroblast activation during wound healing is to re-establish tissue architecture and homoeostasis [68, 69, 87]. Myofibroblasts are responsible for synthesis, arrangement, and remodeling of ECM *via* the secretion of matrix-degrading enzymes (e.g., matrix metalloproteinases MMPs), their inhibitors, and contractility enhancement [68, 69, 88, 89]. The plethora of secreted factors allows myofibroblasts to regulate the phenotype and thus function of other cell types [68, 69, 90] in order to re-vascularize the healing tissue, stimulate motility and proliferation/differentiation balance, and impose inflammatory response, and to secrete and absorb metabolites to rebalance the tissue niche [68, 69, 91]. A key functional difference between fibroblasts and myofibroblasts involves the cell secretome, which in myofibroblasts is characterized by increased release of signaling molecules including growth factors, cytokines, interleukins, matrix proteins, and proteolytic enzymes [68, 69, 92]. Due to their function, fibroblasts and particularly myofibroblasts are subjected to strict control mechanisms. Under physiological conditions, when the process of healing is completed, the number of myofibroblasts markedly decreases [93, 94]. The differentiation can sometimes be reversed or myofibroblasts enter a quiescent or senescent state [68, 95]. If myofibroblasts escape these protective mechanisms, either due to the continuous stimuli like during chronic inflammation or due to intracellular misregulations, persistent FMyoT will lead to proliferation/differentiation impaired balance, self-activation, increased protein deposition, and development of pathological events as fibrosis and cancer [8–13, 68]. Thus, FMyoT can be assumed a cellular hallmark of the pathological state of the tissue [68]. For many years, it has been assumed that myofibroblasts derive exclusively from local fibroblasts. Nowadays, it has become apparent that their origin is multiple including epithelial-myofibroblast transition (EMyoT) [12, 13], mesenchymal stem cell differentiation, and endothelial-mesenchymal transition (EndMT) [96, 97].

### Cancer-associated fibroblasts (CAFs)

Activated fibroblasts and myofibroblasts accompanying tumors are called CAFs. CAFs belong to the principal constituents of the tumor stroma, responsible for tumor microenvironment shaping [10, 97–99]. In contrast to normal fibroblasts and myofibroblasts, CAFs become constantly activated and neither revert to a normal phenotype nor undergo apoptosis and elimination [100, 101]. They contribute to tumor cell proliferation, EMT, invasion, and metastasis *via* secretion of various growth factors, cytokines, and chemokines like stromal-cell-derived factor 1 (SDF-1), and remodeling of ECM proteins [9–13, 94–105]. CAFs were also shown to mediate cancer-related inflammation by secreting numerous pro-inflammatory and tumor-promoting factors [95, 96, 101, 105, 106]. Moreover, under the control of a variety of stroma-modulating factors, the cancer cells themselves generate a permissive microenvironment favoring further tumor development and tumor cell invasion indicating the existence of paracrine interactions between tissue cells participating in the formation of GC loci [95–97, 104–106]. Importantly, the tumor stroma, which consists of CAFs, ECM, and vascular endothelial and immune cells, acts as a barrier promoting tumorigenesis and limiting drug delivery [99–105, 107, 108].

## CAF sources

### Fibroblasts and myofibroblasts

It has been initially proposed that the majority of CAFs originate from the local activated myofibroblasts and fibroblasts [107, 108]. The selected differentiation markers distinguishing fibroblasts, myofibroblasts, and CAFs are presented in the Table 1. Such activation engages multiple signaling pathways triggered by growth factors and cytokines (such as TGF-β1, HGF, osteopontin (OPN), IL-1β, IL-6, IL-8, and SDF-1), glucose metabolism reprogramming, ROS reactive oxygen species (ROS), and hypoxia-inducible factor-1α (HIF-1α) [103, 108]. *Hp* is one of the potent factors able to elicit above pathways and factors. It has been shown that *Hp* infection in the stomach of Mongolian gerbils and mice induces chronic gastritis and atrophy accompanied with a shift from gastric to intestinal phenotype as well as increased number of α-SMA-positive myofibroblasts in the mesenchyme of both cancerous and non-neoplastic mucosa [89, 108, 109]. As mentioned previously, it has been also shown that *Hp* reaches pits of gastric glands and interacts with stroma components including fibroblasts [6, 8–10, 95–98]. Necchi et al. [6] identified the presence of *Hp* in the intraepithelial intercellular spaces, underlying lamina propria, and stromal tumor stroma. Additionally, Kuwada and coworkers [14] studied 71 malignant GI stromal tumor (GIST) cases of which 62.9% were *Hp* positive compared to 65 non-GIST controls, of which 30% were *Hp* positive. Their study utilized PCR detection of *Hp* infection in GI tissue specimens with respect to various *Helicobacter* species, including *H*. *heilmannii*. In the regression analyses performed, GIST cases showed large and significant odds ratios for *Hp* infection detected by PCR compared with controls. Thus, their results strongly suggest the association between *Hp* infection within mesenchymal compartment and GIST. In their study, *Hp* genes were detected in gastric and extragastric GISTs which is consistent with other studies [14, 107, 109, 110]. A considerable number of GISTs were also positive for myofibroblast marker α-SMA, which points to the direct influence of *Hp* on fibroblast activation [14]. Indeed, such interactions have been proposed, suggesting that *Hp* interacts with several other cells beyond its direct effect on gastric epithelium [6, 8–10, 13, 95–98, 107, 111]. Our group has shown that *Hp* can directly interact with rat and human gastric fibroblasts triggering FMyoT and activating them towards CAFs [8–13]. Our mechanistic *in vitro* study has revealed that *Hp* induced strong upregulation of TLR2 and TLR4 mRNAs in fibroblasts already after 3 h of infection, which strongly suggests TLR4 and TLR2 contribution to the early immune protective response against *Hp* and fibroblast activation as reported by other investigators [112–114]. Simultaneously, we have noticed the significant increase in fibroblast expression of NFκB RelA subunit followed by decreased Iκβ [11]. These observations are in keeping with the evidence that binding of TLR2 and TLR4 to *Hp*-derived components caused the recruitment of an adapter molecule, myeloid differentiation primary response 88 (MyD88), leading to the activation of mitogen-activating protein kinase (MAPK) and NFκB which resulted in the expression of proinflammatory cytokines and chemokines [113, 115–118]. Additionally, TLR4 and TLR9 have been shown to act through STAT3-dependent mechanisms [119, 120] also in *Hp*-activated gastric fibroblasts (*Hp*-AGFs) [11]. Accordingly, reciprocal NFκB activation and deregulation of JAK/STAT3 signaling pathway can lead to inflammation and development of GC [119–123]. Next to TLRs, also the major *Hp* cytotoxin CagA can directly utilize NFκB and STAT3, in either a phosphorylation-dependent or phosphorylation-independent manner [124, 125] or through the interaction with TNF receptor–associated factor 6 (TRAF6) and TGF-β-activating kinase 1 (TAK1) [19, 25, 64], leading to the transformation from chronic inflammation to cancer [113, 124, 125]. Additionally, it has been shown that phosphorylated CagA enhanced SH2 containing protein tyrosine phosphatase-2 (SHP2) binding activity leading to activation of ERK/MAPK signaling pathway [125–127]. The nature of the interactions between NFκB and STAT3 includes the overlap of downstream genes, physical interactions [128, 129], and inducer-effector positive feedback loops utilizing interleukins like IL-6 and growth factors, e.g., EGF or HGF. All these interactions have been shown to play the central role in the dialog between the malignant cell and its microenvironment [128, 129]. Consequently, CAFs including *Hp*-AGFs mediate cancer-related inflammation by secreting numerous pro-inflammatory and tumor promoting factors including IL-6, IL-8, SDF-1, TGF-β, EGF, and HGF [4, 9–13, 95–98, 102]. Other downstream targets of STAT3/NFκB interactions: SNAIL, ZEB, and TWIST, were initially described as EMT-inducing transcription factors (EMT-TFs) [130]. However, beyond EMT, they are also involved in the control of global plasticity programs affecting cell stemness and fate [131] and their expression is subjected to strict restrictions [128, 132, 133]. Consequently, the acquisition of EMT-TF expression by activated fibroblasts provides them with additional properties. It has been shown that beginning from the early epithelial tumor stage, SNAIL1 expression is restricted to tumor and tumor stroma cells. Additionally, the SNAIL1 expression positively correlated with the presence of metastasis and lower survival of patients [128, 133, 134]. Loss-of-function experiments in cell culture and mice confirmed that SNAIL1 expression is necessary for driving the CAF-specific secretome [35, 78, 133–135] responsible, e.g., for the promotion of tumor cell invasion [31, 32, 78]. Consequently, *Hp*-induced NFκB/ STAT3 activation resulted in TGF-β-dependent SNAIL1 upregulation in fibroblasts [8–13]. SNAIL1 expression induced a rapid increase in auto- and paracrine TGF-β-induced RhoA activity which led to cytoskeletal rearrangements in fibroblasts and α-SMA incorporation into the stress fibers [10, 134] in addition to other effects exerted through the activation of SMAD-dependent and SMAD-independent pathways [103, 136–139]. Nevertheless, SNAIL1 expression has been also shown to correlate with FMyoT leading to fibrosis [136, 140]. Thus, the additional presence of TWIST has been linked to GC development. The simultaneous expression of SNAIL1 and TWIST1 in stromal cells, particularly in the diffuse type of GC, has been associated with clinical and histopathological outcomes indicating disease progression [133, 138, 139], while the negative expression of these EMT-TFs has predicted a better outcome of the GC patients [133, 141]. Thus, TWIST1 has been proposed to be a novel marker that distinguishes CAFs from normal fibroblasts and importantly from myofibroblasts [104]. It has been shown that IL-6 eliciting reciprocal IL-6/NFκB/STAT3 activation [142] is sufficient to induce TWIST1 expression in normal cultured fibroblasts resulting in their activation into CAFs [11–13, 133, 143]. Importantly, IL-6 was also found to upregulate c-MET expression, with further cooperation of HGF and IL-6 in enhancing TWIST-dependent CAF phenotype [133, 144]. In line with this evidence, the increased TWIST expression in *Hp*-AGFs could have been the consequence of direct CagA-induced NFκB/STAT3 activation leading to increased release of several procarcinogenic factors, i.e., SDF-1, IL-8, TGF-β, and also IL-6 and HGF [9–13] multiplicated by their subsequent autocrine action. Thus, the targeting NFκB/STAT3-dependent TWIST activation offers a great promise as one of the possible targets in cancer pharmacotherapy. It has been also shown that ZEB1 expression in CAFs and cancer cells was associated with poor prognosis [133, 145] while ZEB2 expression with metastasis [133, 146]. Consequently, ZEB1 inactivation in stromal fibroblasts suppressed tumor initiation, progression, and metastasis associated with reduced ECM remodeling, immune cell infiltration, and decreased angiogenesis [133, 146, 147]. However, further reports showed expression of ZEB1 and ZEB2 also in non-tumorigenic activated fibroblasts. It has been demonstrated that ZEB1 may participate in excessive fibrotic events by activating α-SMA promoter and inducing FMyoT resulting in fibrosis and scarring [133, 148–150], which may link ZEB expression with GC-associated fibrosis. Interestingly, we have found ZEB mRNA upregulation in *Hp*-AGFs [11]. EMT-TFs have been also shown to modulate the P53 pathway in cancer cells leading to hyperproliferation and providing chemoresistance to CAFs, an event that likely promoted CAF survival, anticancer therapy resistance, and possibly recurrence [103, 151, 152]. We have also observed that HIF-1α upregulation was an early event in *Hp*-induced fibroblast activation [8]. HIF-1α is a key factor responsible for the regulation of cell adaptation to infection-induced hypoxia [153, 154]. It has been shown that the reactive oxygen species (ROS) induced by CagA^+^ *Hp* or CagA itself can stabilize HIF-1α, an event responsible for its increased expression [155] and subsequent fibroblast activation [8, 153, 154]. HIF signaling is essential for normal cell adaptation to the change in oxygen homeostasis. However, it also plays a key role in the growth of solid tumors, which contain poorly vascularized regions, and its expression correlates with more aggressive phenotype and therapeutic resistance [129, 153, 156]. Recently, Shen and coworkers [157] have shown that *Hp* stimulated JAK/STAT1 signaling in gastric fibroblasts leading to increased expression of VCAM1, which subsequently interacted with integrin αvβ1/5 in GC cells facilitating tumor invasion both *in vitro* and *in vivo*. Finally, our group has reported that *Hp*-AGF induced EMT type 3 responsible for procarcinogenic, proinvasive, and importantly propluripotent reprogramming in normal rat gastric epithelial cells and normal human keratinocyte line HaCaT [9–13]. We have also observed EMT type 3 combined with increased invasiveness and propluripotent reprogramming in human gastric adenocarcinoma AGS cells and in human colon adenocarcinoma HT29 cells [13]. These observations point to a potent and universal cancerogenic action of *Hp*-AGFs.

**Table 1:** Selected cell markers characterizing normal fibroblasts, myofibroblasts, and cancer-associated fibroblasts (CAFs)

Cell markers	Fibroblasts	Myofibroblasts	CAFs
N-Cadherin	+	+	+
FSP1	+	+	+
Stress fibers	F-Actin stress fibers	α-SMA-positive stress fibers	α-SMA-positive stress fibers or dispersed α-SMA distribution
α-SMA/ACTA2	**-**	+	+
Vimentin	+	+	+
Collagen type I	+	↑	↑↑
Collagen type III	+	↑	↑↑
TGF-β	+	↑	↑↑
HGF	+	↑	↑↑
EGF	+	↑	↑↑
SDF-1	+	↑	↑↑
IL-8	+	↑	↑↑
IL-6	+	↑	↑↑
Extra domain A fibronectin (EDA–FN)	**-**	+	↑
Fibronectin	+	↑	↑↑
TNC	**-**	+	+
Periostin	**-**	+	↑↑
FAP	**-**	**-**	+
Snail	**-**	+	+
Twist	**-**	**-**	+
Zeb	**-**	+	+

The magnitude of increased expression is labeled as ↑ or ↑↑ and the presence or the absence of these markers is denoted by either + or − (based on the following references: [8–13, 42, 69, 70, 72, 73, 75–81, 83, 84, 89–102, 104, 128, 134, 135, 143]

### Mesenchymal stem cells

Mesenchymal stem cells (MSCs) are multipotent, adult stem cells, found in several different tissues [157]. They can migrate across tissues and differentiate into a variety of cells, depending on the surrounding microenvironment [132, 139, 157–159]. The MSCs have been found in the sites of tissue injury, supporting their important role in regeneration processes, e.g., in the mechanism of wound healing. Additionally, MSCs derived from the bone marrow (BM-MSCs) or adjacent normal tissues are recruited to the sites of inflammation and inflammation-associated cancer [158, 159] also during *Hp* infection [19, 22, 51, 132, 139, 157–160]. It has been shown that *Hp*-induced epithelial infection can assure direct homing of MSCs into the gastric mucosa where they reprogram towards CAFs [22, 50, 51, 157–160]. Consequently, BM-MSCs repopulated gastric mucosa of mice with intestinal-type adenocarcinomas resulting from chronic infection with different *Helicobacter* strains [22, 51]. Werner and Hoffman [19] have underlined that in contrast to *Hp*-induced inflammation, which is an early event, bone marrow–derived cells (BMDC) repopulation of gastric glands is a late event. Varon and coworkers have shown that in *Hp*-infected C57BL/6J female chimeric mice engrafted with BMDCs from the male donors, about 25% of the dysplastic lesions included BMDCs [51]. Consequently, multiple lines of evidence suggest that significant proportion of CAFs within tumors originates from BM-MSCs [19, 161]. Shi et al. [162] showed that GFP-labeled BM-MSCs homing gastric mucosa during *Hp* infection in mice differentiated in a thrombospondin-2 dependent fashion both into pan-cytokeratin-positive epithelial cells and α-SMA^+^ CAFs [162]. They subsequently performed the FISH analysis of gastric tissue from the woman with acute myeloid leukemia, chronic gastritis, and *Hp* infection, who received a bone marrow transplant from a male donor. The FISH analysis showed Y-chromosome^+^ cells derived from the man bone marrow transplant. The further analysis of immunofluorescence confirmed that Y-chromosome^+^ cells were positive for BM-MSC markers. Thus, the authors concluded that MSCs participate in the development of chronic *Hp*-associated GC by differentiating into both gastric epithelial cells and CAFs [162]. The BM-MSC recruitment process has been shown to involve the upregulation of proinflammatory cytokines and growth factors (e.g., TGF-β, IL-1β, TNF-α, and IL-6) as well as chemokines, such as SDF-1 derived among others from activated fibroblasts [9, 11, 161, 162].

### EMT

EMT is a biological process in which polarized epithelial cells lose the adherence and tight cell-cell junctions and acquiring motile, mesenchymal phenotype. EMT types 1 and 2 are essential for numerous developmental and regenerative processes including wound healing, while EMT type 3 is a prerequisite for tumor promotion and progression allowing normal epithelial and tumor cells to acquire invasive properties and develop metastatic growth characteristics [12, 13, 15, 70, 95, 96, 98, 163, 164]. Importantly, EMT type 3 is also associated with the acquisition of pluripotency and stem-like cell characteristics [12, 13, 165–167]. The selected differentiation markers distinguishing normal epithelial cells from those that have undergone profibrotic EMT type 2 and procarcinogenic EMT type 3 are presented in Table 2. As summarized by Baj and coworkers [168], *Hp* can activate a plethora of signaling pathways leading to EMT. The injection of the CagA into the cell *via* the T4SS activates NF-κB signaling pathway which then involves extracellular regulated kinases ½ (ERK-1/2) activation [168–171], eliciting the conformational changes of the cytoskeleton enhancing the EMT process [172, 173]. Tyrosine-phosphorylated CagA interacts with protein tyrosine phosphatase 2 (SHP-2), also inducing the progression from the settle into motile, so-called hummingbird phenotype [174, 175]. It has been proposed that CagA enhances EMT *via* the stabilization of SNAIL protein mainly by the reduction of glycogen synthase kinase-3 (GSK-3) activity [176]. CagA^+^ *Hp* strains were shown to induce higher expression of SNAIL and ZEB-1 followed by mesenchymal markers such as vimentin, and the stem cell marker CD44 [177, 178]. Molina-Castro et al. [179] showed that infection by CagA^+^ *Hp* upregulated the expression of both Yes1-associated transcriptional regulator 1 (YAP1) and large tumor suppressor 2 (LATS2), the components of the hippo signaling pathway in gastric epithelial cells. Importantly, an overexpression of the oncogenic YAP1 has been associated with EMT-related aggressiveness and poor prognosis [180]. EMT can be also triggered by *Hp*-induced elevation of TNF-α-inducing protein (Tipα protein), which binds to the cell surface nucleolin [181]. By combining with the nucleolin receptor, Tipα induces mesenchymal phenotype switch leading to cancerogenesis [182]. It has been also shown that *Hp* initiates EMT *via* the upregulation of ZEB1 and microRNA (miR)-200 [183]. Finally, TGF-β has been considered one of the most potent among EMT inducers. Recent studies have shown that the alveolar epithelial type 2 cell line RLE-6TN treated with TGF-β1 is converted into myofibroblasts through the Ras-ERK pathway [99]. This finding is consistent with the results obtained by our team, showing that the secretome from *Hp*-AGFs rich among others in HGF, IL-6, and TGF-β triggered EMT-type 3 in both normal gastric epithelial cells and cancer cells. These events were attributed to TGF-β1 release by *Hp*-AGFs/epithelial cells, which modulated TGF-βR1/R2-dependent signaling, consequently prompting pro-CSC-like phenotype, and providing the basis for EMT type 3/MET flexibility [13]. We have also shown the important role of TWIST expression in the mechanism of EMT type 3–related epithelial reprogramming [12, 13].

**Table 2:** Selected cell markers characterizing normal epithelial cells, epithelial cells that underwent EMT type II or EMT type III

Cell markers	Epithelial cells	EMT type II	EMT type III
ZO-1	+	**-**	**-**
E-Cadherin	+	**-**	**-**
Occludin	+	**-**	**-**
Claudin	+	**-**	**-**
Desmoglein	+	**-**	**-**
Desmocollin	+	**-**	**-**
Cytokeratins	+	**-**	**-**
N-Cadherin	**-**	+	+
Vimentin	**-**	+	+
FSP1	**-**	+	+
Stress fibers	**-**	α-SMA-positive stress fibers	Broad diversity of actin cytoskeleton organization, F-actin stress fibers present or absent, dispersed α-SMA distribution
α-SMA/ACTA2	**-**	+	+
FAP	**-**	**-**	+
TGF-β	**-**	**-**	+
HGF	**-**	**-**	+
Snail	**-**	+	+
Twist	**-**	**-**	+
Zeb	**-**	+	+
MMPs	**-**	↑	↑↑
Phenotype	Differentiated	Temporarily transdifferentiated	Pro-pluripotent/flexible

The magnitude of increased expression is labeled as ↑ or ↑↑ and the presence or the absence of these markers is denoted by either + or − (based on the following references: [9–13, 78, 84, 86, 100, 104, 105, 117, 119, 121, 128, 130–133, 136, 142]

### EndMT

EndMT was first observed during heart formation in embryonic period due to the increased tissue level of TGF-β [99, 184, 185]. Basically, the EndMT is a process during which cells lose their polarity and expression of endothelial markers acquiring mesenchymal phenotype associated with fibrosis as well as with cell invasive and migratory properties [185, 186]. Zeisberg et al. demonstrated that CAFs originated from the vascular endothelial cells [185] and similar phenomenon has been shown to occur within the stroma of different cancers [186, 187]. It is not excluded that EndMT may also occur during gastric *Hp* infection. As possible sources of CAFs, smooth muscle cells and adipocytes have been also postulated [188].

## The influence of CAFs on gastric stem cell niche and cancer development

The proliferative activity and the acquisition of particular cell fates are coordinated by a small number of conserved signaling pathways, most notably the Wnt/β-Catenin, BMP, Notch, and TGF-β pathways [17, 19, 73]. Accordingly, myofibroblasts and particularly their activated counterparts have been found to express a variety of genes encoding for both activators and inhibitors of the pathways involved in the regulation of epithelial stem cell niche [17, 19, 73, 189].

### Wnt signaling pathway

**Fig. 2:**
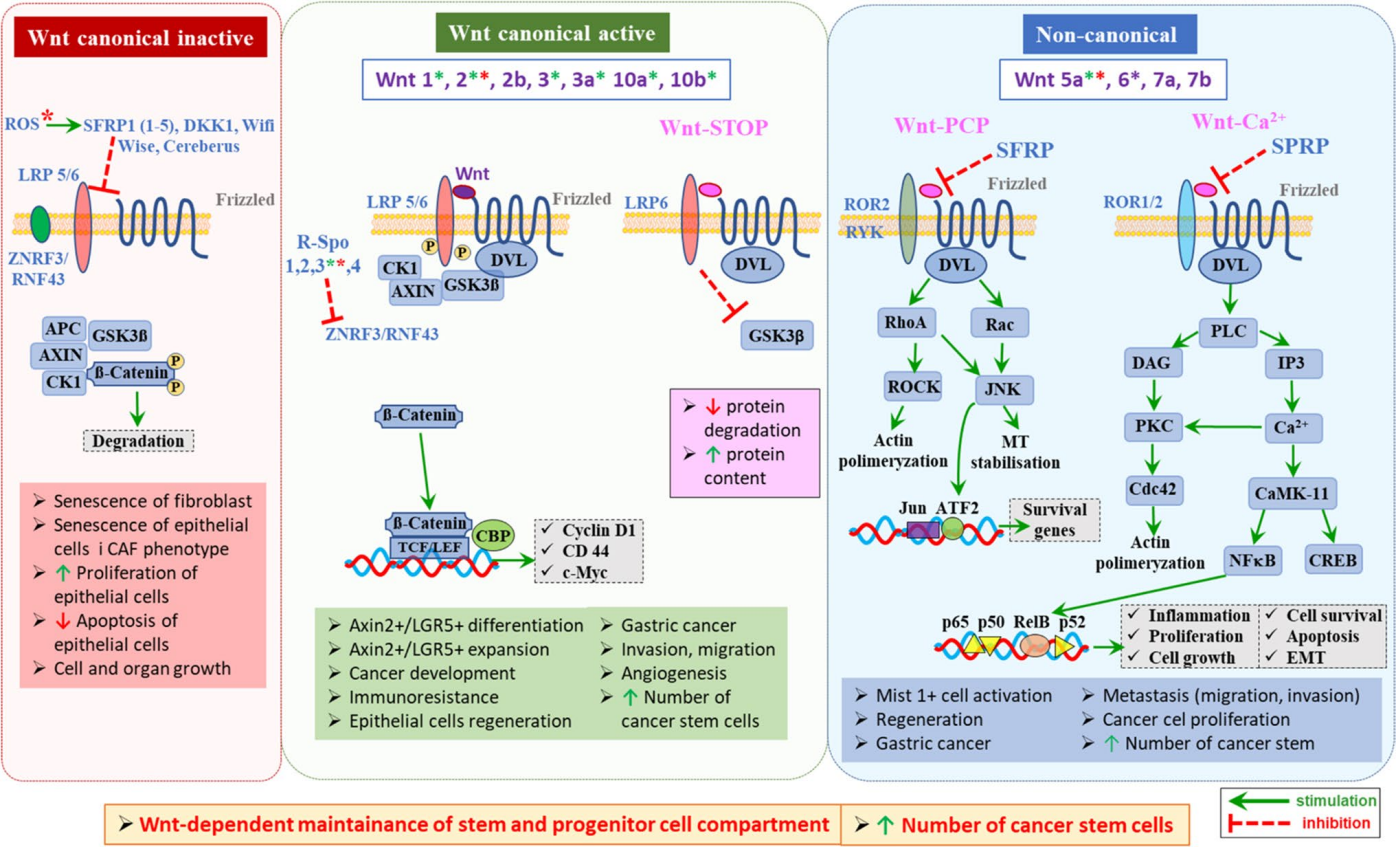
The participation of canonical and non-canonical Wnt/β-catenin signaling pathway in stem/progenitor cell maintenance and GC development. The canonical pathway is activated by increased release of Wnt canonical ligands and sustained by RSPO-induced ZNRF3/RNF43 ubiquitin ligases inactivation. This pathway is essential for determining the cell fate, proliferation, and self-renewal of both stem and progenitor cells by, e.g., regulation of the transcription of Wnt target genes including CD44, cyclin D1, and c-Myc, promoting regeneration of epithelial cells. Non-canonical pathway is activated by increased release of non-canonical Wnt ligands. It leads to planar cell polarity (PCP) signaling responsible for cell cytoskeleton regulation and Wnt/calcium signaling responsible for the regulation of intracellular calcium concentration, as well as CREB and NFκB transcription factors involved in the regulation of inflammatory response. Another non-canonical pathway that might be triggered is Wnt/STOP pathway serving for cellular divisions. Green asterisk denotes the factors that are upregulated during *Hp*-positive GC. Red asterisk denotes the factors released by activated fibroblasts/CAFs during *Hp* infection. ROS, reactive oxygen species

Wnts were reported as ubiquitous instructive signals during development which direct cell fate and patterning [29, 190]. Wnt signaling pathways use either paracrine or autocrine communication. Three Wnt signaling pathways have been characterized: the canonical Wnt pathway leading to the regulation of gene transcription, the non-canonical planar cell polarity (PCP) pathway which regulates cell cytoskeleton, and the non-canonical Wnt/calcium pathway that regulates calcium homeostasis inside the cell. All three pathways are activated by the binding of Wnt ligands to heterodimeric receptor complex of Frizzled receptors (FZD1-10) and their co-receptors, low-density lipoprotein receptor-related protein 5/6 (LRP5/6), and receptor tyrosine kinase-like orphan receptor 2 (ROR2), and related to receptor tyrosine kinase (RYK), to initiate either β-catenin-dependent (canonical) or β-catenin-independent, non-canonical signaling [29, 190–192]. The Wnt ligands have been classified into canonical Wnt ligands, which induce a β-catenin-dependent pathway (WNT1, 2, 3, 8a, 8b, 10a, and 10b), and non-canonical Wnt ligands, which induce β-catenin-independent pathways (WNT4, 5a, 5b, 6, 7a, 7b, and 11) [193, 194]. The canonical Wnt signaling pathway is essential for determining the cell fate, proliferation, and self-renewal of both stem and progenitor cells [191, 195]. This pathway depends on the stabilization of β-catenin involved in the cell-cell interactions within adherens junctions, necessary for cell growth, adhesion between cells, and contact inhibition and thus for maintenance of epithelial cell layers and barriers [29, 190–198]. The canonical Wnt pathway is activated through the binding of canonical Wnt ligands (such as Wnt3a). This event leads to dimerization of the Fzd receptor and LRP5/6 co-receptor. Then, LRP receptors are phosphorylated by GSK3β, which is responsible for the releases of β-catenin from the AXIN complex. β-Catenin then translocates to the nucleus, and binds to members of the T cell factor/lymphoid enhancer factor (TCF/LEF) family and CREB-binding protein (CBP) acting as transcription co-activator. This latter step is involved in the modulation of transcription of Wnt target genes including *CD44*, *cyclin D1*, and *c-Myc* [103, 191, 193, 199, 200]. These target genes are engaged in the promotion of epithelial cell regeneration through focal adhesion kinase (Fak) and Akt/mTOR signaling (Fig. 2) [193, 201]. In the absence of canonical Wnt signaling, the pathway is inactivated through a negative feedback mechanism, involving β-catenin destruction complex. This complex targets β-catenin for proteasomal degradation, and the plasma membrane ZNRF3/RNF43 ubiquitin ligases, which function through promoting ubiquitination and degradation of Wnt receptors [199, 202, 203]. Binding of WNT5a, WNT7a, WNT8a, or WNT11 (non-canonical Wnts) to Fzd receptors and their co-receptors ROR2 and RYK initiates PCP leading to Rho-dependent ROCK1 activation and cytoskeletal rearrangement (Fig. 2) [191, 195, 203]. The disparity in Wnt ligand/receptor complexes was shown to allow for variation in the signal transmission, resulting in the activation of genes, such as *c-jun*, *Cdc42*, and *dvl* [192]. Alternatively, the PCP can also be activated upon binding of syndecan 4 (SDC4) and R-SPO to FZD7, transmitting WNT5a (non-canonical Wnt)-induced internalization of the ligand/receptor complex [172, 173, 191]. Initiation of Wnt/Ca^2+^ signaling through Fzd/ROR1/2 interaction activates inositol 1,4,5-triphosphate (IP_3_) and 1,2-diacylglycerol (DAG) and induces Ca^2+^ release from endoplasmic reticulum. DAG together with Ca^2+^ activates PKC and Ca^2+^ activates calmodulin-dependent protein kinase II (CaMKII). In turn, both kinases activate CREB and NFκB transcription factors [173, 191, 204]. Recently, a novel arm of non-canonical Wnt signaling known as the Wnt/STOP pathway (Wnt-dependent stabilization of proteins) was discovered (Fig. 2) [195]. Canonical Wnt signaling peaks during the G2/M phase of the cell cycle as LRP6 is primed by Cyclin Y/CDK14, and at this point, activation of β-catenin-independent stabilization of proteins is seen, leading to inhibition of GSK-3β activity. This prevents degradation of multiple proteins, resulting in increased cell protein content, the prerequisite for cellular division [191, 195]. Notably, it has been shown that mesenchymal cells including fibroblasts and myofibroblasts are a rich source of Wnt ligands [193]. It has been reported that blockage of the Wnt ligands secreted by subepithelial telocytes impairs epithelial renewal and alter intestinal integrity, leading to loss of stem and progenitor cells [193, 204, 205]. In the stomach and intestine, there is strong evidence for Wnt (WNT5a and Wnt2b) gradient responsible for the maintenance of stem and progenitor cells and their proper proliferation. This gradient is responsible for the regulation of L-GPC and T-GPC fates at the gland bottom. Additionally, Lgr5 and Troy have been established as the Wnt target genes [17, 19, 65, 176, 205]. Importantly, the proper ratio of Wnt to SFRP1 release by subepithelial telocytes has been shown to regulate the compartmentalization of stem and progenitor cells [205]. Degirmenci et al. [206] showed that glioma-associated oncogene (GLI1)-expressing mesenchymal cells formed the essential Wnt-secreting niche for supporting the colon stem cells. The specific blockage of Wnt ligands provoked the loss of stem cells, which resulted in alterations of internal epithelium integrity and eventually epithelial death [193, 206]. In the isthmus region of the gastric corpus, the Wnt signaling pathway has been shown to be essential in maintaining the undifferentiated state of progenitor cells, while in the antrum its activation increased the number of progenitor cells [17, 59]. Hayakawa and coworkers found that stroma cells residing in the gastric epithelium provide an additional environment for gastric corpus stem cell niche partly through the production of WNT5a [27]. WNT5a was also shown to activate Mist1^+^ isthmus stem cells in the corpus [17, 27]. Consequently, the lack of WNT5a caused several alterations in the GI tract [206]. WNT5a has also been found to activate the regeneration of the intestinal crypts through TGF-β signaling [17, 86, 193]. The activation of Wnt pathway in gastric stem cells has been reported to associate with gastric carcinogenesis in mice [59, 61]. As summarized by Koushyar, the genomic analysis identified 46% (range 43–48%) of gastric tumors exhibiting deregulation of the Wnt/β-catenin pathway. Also, several Wnt ligands have been shown to be upregulated, including WNT1, WNT2b, WNT5a, WNT6, and WNT10a [191]. Moreover, in 13/15 GC cell lines, nuclear localization of endogenous β-catenin, leading to subsequent increase in TCF/LEF transcriptional activity, has been observed [191, 207]. High protein expression of the non-canonical WNT5a ligand was observed in 71 out of 237 primary GC patient samples (both intestinal and diffuse types) and that expression positively correlated with the depth of invasion and the degree of lymph node metastasis [208]. Furthermore, an *in vivo* xenograft model showed that the injection of metastatic GC cells with stable WNT5a knockdown into the spleen of nude mice significantly decreased the number of liver metastatic nodules when compared to control GC cell lines [208, 209]. The WNT5a has also been associated with GC cell migration and invasion *in vitro* [208, 210]. Importantly, Wnt ligands have been identified as one of the main factors responsible or CAF-epithelial cell communication. The WNT2 protein secreted by CAFs enhanced cell invasion, migration, and angiogenesis in colorectal cancer [103, 211]. Notably, WNT5a was upregulated in CAFs, and its inhibition suppressed GC cell growth and migration [103, 212]. On the other hand, it has been shown that both oversecretion and absence of Wnt ligands exert procancerogenic action [213–216]. Interestingly, reactive oxygen species (ROS) activates canonical β-catenin-dependent Wnt signal pathway, which is responsible for upregulation of c-Myc at the invasive front enriched in cancer stem-like cells [217, 218]. Importantly, Wnt/β-catenin signaling pathway is considered to act as the most prominent axis associated with *Hp*-induced hyperproliferation [208–211]. The molecules responsible for Wnt signaling pathway inhibition are subdivided into secreted frizzled-related proteins (SFRP1–5), Dickkopf family proteins (DKK1-4), Wnt inhibitory factor (Wif1), Wise/SOST, and Cerberus. Among these proteins, the SFRPs, Wif1, and Cerberus are able to sequester Wnt agonists in the extracellular environment, while DKK proteins and Wise/SOST compete with Wnt agonists and prevent their binding with the receptor LRP5/6 [193, 219]. It has been shown that DKK1 and SFRP factors possessed strong ability for senescence induction. Different stressors such as DNA or oxidative damage–derived ROS which are also resulting from *Hp* infection can induce SFRP1 secretion from human primary fibroblasts. This effect is subsequently responsible for autocrine senescence in stressed fibroblasts and paracrine senescence in epithelial cells [220]. Additionally, it has been reported that both low and high level of Wnt influenced fibroblast phenotype. Low level of Wnt induced inflammatory CAF (iCAF) phenotype while high level of Wnt induced myofibroblastic CAF (myoCAF) phenotype [213]. Joesting et al. [214] have shown elevated secretion of SFRP1 in prostatic tumor stroma that can be associated with the overexpression of SFRP1 in tumor-committed prostatic epithelial cells. Furthermore, treatment of a human prostatic epithelial cell line with SFRP1 led to increased proliferation, decreased apoptosis, and decreased signaling through the Wnt/β-catenin pathway *in vitro* and increased proliferation *in vivo*. At the same time, treatment of developing prostates with SFRP1 in cell culture led to increased organ growth [214].

### R-spondins

The natural antagonists of ZNRF3/RNF43 are RSPO 1–4 [203]. RSPO bind to LGR4/5/6 receptors, leading to inactivation of ZNRF3/RNF43 ubiquitin ligases and stabilization of Frizzled receptors. This effect led to the potentiation of Wnt/β-catenin signaling particularly within stem cell compartment [221]. It has been shown that organoids from the gastric corpus resembled glands only in the presence of Wnt and RSPO1 [35]. Siegal and coworkers showed that another member of RSPO family, RSPO3, was released by myofibroblasts of the stroma adjacent to the stem cell compartment directly beneath the glands [221]. This finding was confirmed in cultured primary antral myofibroblasts [221]. It has also been shown that RSPO3 induced differentiation of AXIN2^+^/LGR5^+^ stem cells into secretory cells with antimicrobial activity, protecting the stem cell compartment against bacterial colonization. In contrast, RSPO3 induced proliferation and expansion of AXIN2^+^/LGR5^−^ cells indicating that myofibroblasts specifically activate gland regeneration through the RSPO3 stimulation of AXIN2^+^/LGR5^-^ cells, while the less proliferative AXIN2^+^/LGR5^+^ L-GPCs appeared to be silenced [221]. Additionally, it has been shown that *Hp* infection enhanced stromal *RSPO3* expression along with the expansion of proliferative AXIN2^+^/LGR5^−^ stem cells and hyperplasia [222]. Consequently, misregulations in RSPO signaling have been implicated in cancer development and immunoresistance [222].

### BMP signaling pathway

**Fig. 3:**
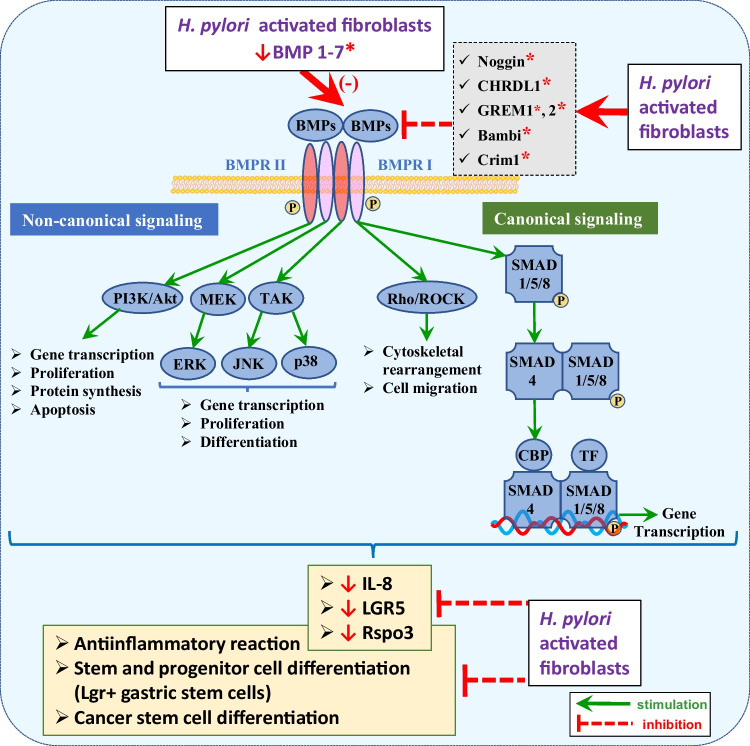
The influence of *Hp*-infected fibroblasts on downregulation of canonical and non-canonical BMP signaling. BMPs are regulatory peptides which through the canonical, SMAD-dependent, and non-canonical, SMAD-independent signaling induce mesenchymal, gastric epithelial, and cancer stem cell differentiation. BMPs have also been reported to evoke anti-inflammatory response by inhibition of IL-8 gene expression. *Hp* infection leads to decreased expression of BMP1 to 7 and increased expression of BMP inhibitors such as CRIM1, CHRDL1, GREM1, and BAMBI, thus diminishing differentiation of stem and progenitor cells leading to hyperproliferation and hyperplasia accompanied with inflammatory reaction. Red asterisk denotes the factors released by activated fibroblasts/CAFs during *Hp* infection

BMPs belong to the TGF-β superfamily of signaling molecules performing the key regulatory functions during development and tissue homeostasis in auto- and paracrine fashion [223]. When activated, TGF-β superfamily molecules act as dimers. They initiate signal transduction through the binding to a Type I or II receptor. Ligand binding leads to the formation of heterotetrameric signaling complex consisting of two Type I and two Type II receptors. The assembly of heterotetrameric complex depends on the ligand specificity [224]. In canonical signaling, the TGF-β superfamily receptors recruit and activate receptor R-SMAD proteins, defined by the presence of a short SSXS activation motif which enables their interaction with Type I receptors [225]. TGF-β superfamily receptors are defined as TGF-β-like or BMP-like depending upon their interaction with one of two R-SMAD groups: SMAD2/3 in TGF-β-like and SMAD1/5/8 in BMP-like signaling. Phosphorylated R-SMAD proteins form a complex with SMAD4 which facilitates their translocation and accumulation in the nucleus. Within the nucleus, R-SMAD–SMAD4 complexes bind directly to DNA and together with other regulatory transcription factors control the expression of numerous genes [224, 225]. In addition to SMAD-related canonical signaling, both TGF-β and BMP receptor complexes recruit and activate a variety of other intracellular signaling mediators [223]. Besides canonical SMAD signaling, TGF-β/BMP ligands have also been reported to signal *via* non-canonical, SMAD-independent pathways. Both BMP and TGF-βs were shown to activate PI3K/Akt as well as TGF-β-activated kinase 1 (TAK1) and MEK leading to ERK, JNK, and p38 kinase activation [224–229] and thus to contribute to the induction of EMT, cell proliferation, and migration (Fig. 3). The ability to elicit a diverse range of cellular responses allows TGF-β and BMP signals to have multiple roles in directing cell fate during development. The human pluripotent stem cells (hPSCs) require the activation or inhibition of TGF-β/BMP signals throughout multiple stages to drive differentiation [223]. BMPs are regulatory peptides mainly secreted by myofibroblast-like cells and involved in mesenchymal stem cell differentiation [227, 229]. BMPs have also been reported to control the differentiation of cancer stem cells [226, 227, 229, 230]. In the stomach, BMP target cells are located in the neck and isthmus [17, 226, 227, 230]. It has been shown that inhibition of BMP signaling in the stomach can lead to perturbations of normal homeostatic mechanisms of the gastric mucosa leading to increased cell proliferation and disturbed differentiation, which result in the development of metaplasia, dysplasia, and neoplasia [17, 227, 229, 230]. Tokabayashi et al. [228] showed that BMPs (BMP-2, BMP-4, and BMP-7) inhibited the expression of the IL-8 gene, a well-established mediator of *Helicobacter*-induced gastric inflammation, after 72 h of incubation in mucus, parietal, and gastric adenocarcinoma cells. This inhibition resulted in the reduced inflammation and restrained dysplastic changes in the gastric mucosa after infection of mice with *Hp* or *H felis*. Consequently, inhibition of BMP signaling led to an enhanced response to inflammatory stimuli and to the accelerated development of dysplastic changes of the gastric mucosa [228]. Culture of myofibroblasts isolated from the entire antrum (including subpopulations from beneath the gland and intraglandular cells) revealed that BMP negatively regulates RSPO3 expression in these cells. These results suggest that under physiological conditions, RSPO3 can be only expressed in the niche lacking BMP signaling and thus is limited to the basal myofibroblasts [230]. Importantly, Jablonska [230] showed that myofibroblast infection with *Hp* increased expression of BMP inhibitors such as CRIM1, CHRDL1, GREM1, and BAMBI and decreased expression of several BMP ligands such as BMP2, BMP6, and BMP7, suggesting enhanced proinflammatory properties of infected myofibroblasts [230]. She has additionally observed reduction for BMP1, BMP3, BMP4, BMP5, and BMP pathway target genes, e.g., of inhibitors of differentiation genes Id1 and Id2 [230, 231]. The Id proteins are expressed by embryonic and somatic stem cells, in which they enhance proliferation and inhibit differentiation [230–234], which may explain the induction and expansion of RSPO^+^ myofibroblasts with decreasing BMP gradient from the base to the stroma along the gland axis [29, 230]. In the context of gastric stem cells, it is also noteworthy that initial low BMP-induced decrease in Id genes can be subsequently turned into their elevated expression after mitogenic stimulation [235]. Thus, besides their crucial role during development, Id-protein expression has been linked to a number of pathologies. Deregulated Id-protein expression has been associated with tumor growth, vascularization, invasiveness, metastasis, chemoresistance, and stemness (summarized by Roschger and Cabrele) [231] and increased ID1 expression has been found in gastric adenocarcinoma [231, 234–236]. This remains in agreement with the previous *in vivo* observations, which identified hyperplasia, hyperproliferation, loss of surface mucous cells, and induction of mucous neck cells resulting from overexpression of BMP inhibitor Noggin or inhibition of BMPR1 receptor in both mesenchymal and epithelial cells [190, 234, 235]. Furthermore, L-GPCs but not WNT target genes such as AXIN2^+^ cells were inhibited by BMP ligands [58, 235, 237], which is consistent with the downregulation of LGR5 in organoids upon BMP2 and BMP4 treatment [235–237].

### TGF-β pathway

**Fig. 4:**
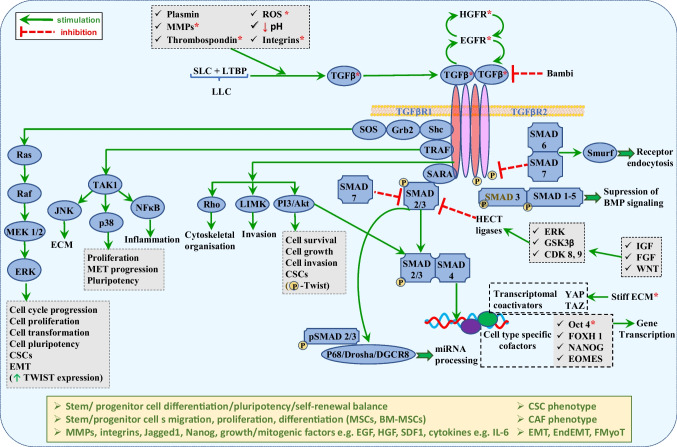
The participation of *Hp*-AGFs in TGF-β canonical and non-canonical signaling. *Hp*-AGFs release increased amounts of TGF-β and stimulate its bioavailability and activation, leading to pathological events including EMT type 3, cell transformation, pluripotency, and overproliferation evoking CSC and CAF phenotypes. In most of the context, active TGF-β signals through a canonical, SMAD-dependent pathway. The retention of R-SMAD and co-SMAD complexes is mediated by transcriptional co-factors such as transcriptional co-activator with PDZ-binding motif (TAZ) and Yes-associated protein (YAP), which can also be activated by stiff ECM produced by *Hp*-activated fibroblasts. The stable binding of SMAD complexes to DNA requires interaction and cooperation with the cell type–specific co-factors FOXH1, EOMES, OCT4, and NANOG, particularly involved in the induction and maintenance of stem cell properties. R-SMADs can regulate miRNA processing by associating with the p68/Drosha/DGCR8 miRNA processing complex. Inhibitory SMADs (I SMAD) such as SMAD6 and 7 bind to activated receptors competing with R-SMAD and recruit the SMURF ubiquitin ligases leading to receptor degradation. Activated R-SMAD proteins could also be degraded by ubiquitination *via* HECT E3 ligases; thus, the duration of TGF-β family signaling integrates with other pathways such as IGF, FGF, and WNT in dictating stem cell homeostasis. The non-canonical, SMAD-independent TGF-β pathways are important effectors for tyrosine kinase receptors. TGF-β activates non-SMAD pathways either directly or through the adaptor proteins. TGF-β can directly activate the Ras/Raf/MEK/ERK/MAPK participating in the regulation of cell cycle progression, cell differentiation, and proliferation. TGF-β receptor complex is also able to activate TAK1, resulting in p38 and JNK and NFκB activation important in pluripotency and MET progression. TGF-β has been also shown to modulate the activities of the small GTPase proteins Rho, Rac, and Cdc42, responsible for the regulation of gene expression and cytoskeleton organization. TGF-β-activated RhoA can activate its downstream targets ROCK and LIM kinase influencing metastasis. TGF-β also induces PI3K/Akt which plays an important role in cell survival, growth, migration, and invasion. Moreover, PI3K/Akt signaling led to phosphorylation and activation of TWIST, thus promoting CSC-like phenotype, survival, and invasion of cancer cells. The reciprocal, potentiating interactions between HGFR-, EGFR-, and TGF-βR-activated pathways have been proposed. TGF-β mediates the migration, proliferation, and differentiation of stem cells into myofibroblasts recruited into the tumor bed. TGF-β is also able to stimulate EndMT and FMyoT. Red asterisk denotes the factors stimulated or released by activated fibroblasts/CAFs during *Hp* infection

TGF-βs1–3 are multifunctional cytokines belonging to the transforming growth factor superfamily. They regulate cell proliferation, migration, and differentiation during embryonic development, and play an essential role in maintaining tissue homeostasis. TGF-β can only exert its biologic function when activated, which can be achieved in response to ECM perturbations. Such perturbations are the frequent event following *Hp* infection particularly regarding *Hp*-induced activation of fibroblasts towards CAFs characterized by the increased production of several ECM components [8–13, 137, 238]. Thus, the TGF-β complex has been postulated to function as a molecular sensor responding through the release of an active ligand [137, 237–239]. Active TGF-β promotes or inhibits cell proliferation in a context-dependent manner. The precise spatiotemporal activation of TGF-β recruits stem/progenitor cells to participate in the tissue regeneration/remodeling process. Consequently, the abnormalities in TGF-β ligand expression, bioavailability, activation, receptor assemblage/stabilization, or post-transcriptional modifications lead to pathogenesis of major diseases. It is exerted either through the recruitment of excessive progenitors or trans-differentiation/reprogramming of resident cells to unfavorable lineage commitment (as seen in auto- and paracrine fibroblast activation and pro-pluripotent EMT type 3 during cancer development and metastasis) [12, 13, 98, 131, 133, 137, 152, 155]. As summarized by Xu et al. [239], TGF-β1 is the most abundant and ubiquitously expressed isoform of TGF-βs. Importantly, TGF-β1-mediated effects are usually restricted to the sites where the active ligand is released from the latent complex stored in the ECM. The precursor TGF-β1 peptide contains signal peptide and TGF-β1 pro-protein which become then processed inside the cell to generate N-terminal latency-associated peptide (LAP) and C-terminal mature TGF-β1. Both LAP and mature TGF-β1 form homodimers *via* disulfide bonds. After secretion, the LAP and TGF-β1 homodimers are non-covalently associated as the small latent TGF-β1 complex (SLC). In most cases, LAP of the SLC covalently associates with a latent TGF-β1 binding protein (LTBP) in the ECM, creating the large latent complex (LLC) that functions as an ECM reservoir of TGF-β, enabling temporal and spatial activation of TGF-β1. This activation requires the release of LLC from ECM and further proteolysis/deformation of LAP to release active TGF-β1 [137, 238, 239]. Accumulating evidence has shown that TGF-β1 can be activated by plasmin, matrix metalloproteinases (MMPs), e.g., MMP-2 and MMP-9, thrombospondin-1, lower pH, and reactive oxygen species [137, 239, 240]. TGF-β1 can also be activated by specific integrins that bind the Arg-Gly-Asp (RGD) sequence of LAPs (Fig. 4). The first proposed mechanism of integrin-based activation requires the participation of MMPs. Integrins are suggested to arrange MMPs, latent TGF-β1, and the TGF-β receptor in the way allowing for the activation of latent TGF-β1 by proteolytical cleavage. Additionally, the integrin-β1-FAK-JNK signaling pathway has been shown to upregulate the expression of both MMP-2 and MMP-9 [239–243]. Consequently, our team has found the increased concentration of TGF-β1 in the secretome of *Hp*-AGFs [10–13]. The TGF-β1-rich secretome induced an increase in the expression of integrins, MMP-2 and MMP-9, in gastric epithelial cells, gastric adenocarcinoma, and colon adenocarcinoma cells. Additionally, these cells acquired the ability to produce and release TGF-β themselves [10–13]. The second mechanism of integrin-based TGF-β activation is associated with forces of cell traction that are directly transmitted to the LLC through the binding of integrins. The cellular contractile force can lead to conformational change of the latent complex releasing TGF-β and/or presenting it to its receptor [137, 239, 241–243]. In most of the context, active TGF-β signals through a canonical (SMAD-mediated) pathway. In canonical pathway, ligand binding to a type II receptor phosphorylates type I partner receptor which then transmits intracellular signaling through the phosphorylation of downstream effector SMADs [137, 244, 245]. Activated R-SMADs form heterotrimers with SMAD4 shared by the TGF-β/activin/nodal and BMP signaling pathways and translocate into the nucleus. Complexes of phosphorylated SMAD2/3 and SMAD4 bind to AGAC or its complement GTCT, known as a SMAD-binding element (SBE) [137, 246, 247]. The increased nuclear retention of R-SMAD and Co-SMAD complexes is mediated by transcriptional co-factors such as transcriptional co-activator with PDZ-binding motif (TAZ) and Yes-associated protein (YAP), which are the downstream effectors of the Hippo pathway. The overexpression of YAP has been associated with aggressiveness and poor prognosis in GC [180]. YAP can also be activated by stiff ECM which is produced, e.g., by *Hp*-AGFs [11, 169, 247–250], and points to TGF-β cooperation with the Hippo pathway in sensing physical environment, cell growth, contact inhibition, and stemness [137]. Protein phosphatases (e.g., PPMA1 or SCPs) can dephosphorylate the R-SMADs, leading to the disruption of the trimer, and eventually turn off SMAD signaling [137, 251]. The DNA-binding affinity of SMAD complexes is weak, and they need to interact and cooperate with other DNA sequence–specific transcription factors to target the specific downstream genes [137, 252–254]. Thus, the availability of cell type–specific co-factors determines the cellular response to TGF-β signaling [17, 137]. Among the co-factors, FOXH1, EOMES, OCT4, and NANOG are particularly involved in the induction and maintenance of stem cell properties [137, 255]. Consequently, we have shown that gastric epithelial cells as well as gastric and colon adenocarcinoma cells cultured in TGF-β-rich secretome from *Hp*-activated fibroblasts were characterized by increased expression of OCT4, which decreased significantly after inhibition of TGF-βR1 activity by its antagonist, SB-43152 [12]. Although most of canonical TGF-β signaling utilizes R-SMADs, not all transcriptional responses require SMAD4 participation. It has been also shown that R-SMADs can regulate miRNA processing in a SMAD4-independent and RNA-sequence-specific manner by associating with the p68/Drosha/DGCR8 miRNA processing complex [17, 137, 256]. TGF-β receptors are regulated by endocytosis [137, 257]. Additionally, inhibitory SMADs (I-SMAD) such as SMAD 6 and 7 (also being TGF-β targets) bind to activated receptors competing with R-SMAD and recruit the SMURF ubiquitin ligases which are responsible for receptor degradation [137, 244–246, 249–254]. Activated R-SMAD proteins could also be degraded by proteasomal ubiquitination *via* HECT E3 ligases such as SMURF1, 2, NEDD4L, and WWP2 [137, 256–260]. R-SMAD proteins contain multiple PY motifs in the linker region [137, 259]. Serine/threonine and proline residues of PY motifs can be phosphorylated by ERK, GSK3, and CDK8 and 9, which enable the interaction with WW domains of HECT E3 and subsequent degradation by the proteasome. These events lead to the termination of transcriptional activity [137, 261, 262]. Thus, as stated by Xu et al., the duration of TGF-β family signaling integrates with other pathways such as IGF, FGF, and WNT [137]. It has been shown that the cross talk between TGF-β superfamily and Wnt signaling pathways has an essential role in dictating stem cell homeostasis in concert with combinatorial activities of other signaling pathways. For example, the cross talk between Nodal/Activin/SMAD2/3, ERK/MAPK, and Wnt/GSK3β/β-catenin pathways which regulates the balance of self-renewal and differentiation status of epithelial stem cells (ESCs) has been described [137, 263]. The SMAD-independent, non-canonical TGF-β pathways are considered important effectors for tyrosine kinase receptors [137, 264, 265]. TGF-β activates non-SMAD pathways through the interactions with the type I/II receptors, either directly or through the adaptor proteins. Nevertheless, the SMAD-mediated downstream gene expression may also activate non-SMAD pathways. TGF-β can directly activate the Ras/Raf/MEK/ERK/MAPK pathway through the interaction between TGF-β receptor complex and ShcA protein. In response to TGF-β, TGF-β type I receptor mediates tyrosine phosphorylation of ShcA, which then recruits GRB2 and SOS to form a complex, initiating Ras activation and consequently ERK/MAPK signaling cascade [12, 137, 264]. It has been proposed that MEK1 and MEK2 participate in the regulation of cell cycle progression, cell differentiation, and proliferation [137, 265]. The Ras-dependent ERK1/2 MAP kinase signaling pathway plays a central role in the control of cell proliferation and is frequently activated in different human cancers. Early studies have shown that expression of activated alleles of MEK1 is sufficient to deregulate the proliferation and trigger the morphological transformation of cell lines [266–268]. Moreover, ERKs have been shown to be vital for CSC tumorigenesis [269]. In concordance, we have observed the significant upregulation of MEK1 and Ras-dependent TWIST activation combined with the acquisition of pro-pluripotent and invasive properties of gastric epithelial cells cultured in the secretome from *Hp*-AGFs [12, 13]. TGF-β receptor complex is also able to activate TAK1, through ubiquitin ligase TRAF6, which has been shown to interact with the TGF-β receptor complex, leading to the activation of p38 and JNK MAPK signaling [19, 25, 64, 137, 225, 230]. P38 MAPK, which activity is significantly increased in GC tissues, has been shown to play an important role in proliferation, MET progression, and establishment of pluripotent phenotype, which are the necessary steps for the development of human iPSCs [270]. Parallelly, it has been shown that inhibition of p38 MAPK activity led to an increase of SMAD2 and SMAD3 which were engaged in the maintenance of pluripotency in hESCs by transcriptional upregulation of NANOG, which then acts *via* the negative feedback loop limiting transcriptional activity of the SMAD2/3 cascade [137, 265, 271]. However, the inhibition of TGF-β/activin signaling or activation of BMP signaling resulted in the loss of pluripotency of both human ESCs and mouse EpiSCs, while activation of TGF-β/activin signaling induced differentiation of mouse ESCs in the absence of LIF (leukemia inhibitory factor), the member of the IL-6 cytokine family [255]. We have shown that limited TGF-β signaling was a necessary condition to maintain pro-pluripotent phenotype of *Hp*-AGF secretome-reprogrammed gastric epithelial cells [12]. TGF-β has been also shown to modulate the activities of the small GTPase proteins Rho, Rac, and Cdc42, responsible for the regulation of gene expression and cytoskeleton organization [137, 272]. TGF-β-activated RhoA can activate its downstream targets ROCK and LIM kinase which overexpression is correlated with metastasis in patients with GC [137, 273]. Another non-canonical TGF-β-induced pathway is PI3K/Akt which plays an important role in cell survival, growth, migration, and invasion [137, 274–276]. Due to the activation of PI3K/Akt signaling, activin A/SMAD2/3 upregulates pluripotency associated genes, e.g., Nanog. In the absence of PI3K signaling, ERK targets GSK3β and thus activates Wnt effectors, and cooperates with SMAD2/3 to promote differentiation which is believed to be important for cell fate decisions during early embryonic development [137, 263]. Additionally, it has been shown that PI3K/Akt signaling led to phosphorylation and activation of TWIST, thus promoting CSC-like phenotype, survival, and invasion of cancer cells [137, 138]. TGF-β may also induce Notch receptor ligands, including Jagged1 (JAG1), which is an important part of molecular machinery regulating cell fate decisions [137, 277, 278]. Notch signaling activates TGF-β signaling pathway potentiating EMT [12, 137, 279] *via* positive feedback loop. However, it has been shown that in certain cell types, e.g., esophageal epithelial cells, Notch signaling may counteract EMT by induction of miR200 which targets ZEB and TGF-β [137, 280]. TGF-β participates in most of the steps through which quiescent stem cells re-enter the cell cycle in response to specific environmental cues and give rise to lineage-specific progenitors which then differentiate to make functional tissue. Thereby, the precise control of magnitude and duration of TGF-β signaling exerted at the level of the synthesis, activation, receptor expression, and the activation and stability of SMAD molecules and other downstream effectors is essential for proper balance between self-renewal and differentiation of stem cells [137, 255, 280]. As brilliantly summarized by Sakaki-Yumoto and coworkers [255], differential expression of all mentioned TGF-β signaling regulators within different stem cells and their niches results in the diverse effects exerted by TGF-β family members on certain types of stem cells. In various cell types, and in embryonic stem cells, SMADs cooperate with master regulators of cell differentiation or pluripotency [137, 255, 280], which has been linked to the expression of “Yamanaka factors” such as OCT4, KLF4, SOX2, and c-MYC [165, 255]. It has been found that “Yamanaka” factors inhibit TGF-β signaling pathway that induces EMT; thus, factors that antagonize TGF-β signaling are believed to enhance reprogramming [281]. However, we have shown that the long-term incubation of gastric epithelial cells in the presence of TGF-β-rich, *Hp*-AGF secretome induced their shift towards propluripotent LGR5^+^/Oct4^high^/Sox-2^high^/c-Myc^high^/Klf4^low^ phenotype, which depended on TGF-β signaling. This phenotype was accompanied by the appearance of both inactive, unmodified form of TGF-βR2 and lower amount of functional, glycosylated form of TGF-βR2. Thus, pro-pluripotent phenotype depended on lowered but still active TGF-β signaling [12]. In concordance with these results, it has been shown that LGR5 and β-catenin expression becomes upregulated in GC cells co-cultured with Tregs or treated with exogenous TGF-β1. Notably, this upregulation was partially inhibited by the TGF-β1 neutralizing antibody or TGF-β1 receptor antagonist SB431542 [282]. It has been demonstrated that Lgr5 is the downstream target of WNT/β-catenin pathway, critical for the promotion of GI cancer proliferation which points to the interaction between TGF-β1 and Wnt signaling pathways especially in the view of the fact that LGR5 is highly expressed in tumor tissues with nuclear accumulation of β-catenin [282–284]. It is corroborative with the findings related to the enrichment of LGR5-positive cells observed in GI tumors [282, 285, 286]. Systematic reviews and meta-analyses have shown that LGR5 is a predictive factor for tumor invasion, metastasis, and resistance to chemotherapy and an indicator of poor prognosis in GC [282, 285, 286]. As mentioned previously, the major pathway induced by tyrosine kinase receptors is the Ras pathway. Cooperation between Ras and TGF-β signaling is particularly important during EMT [137, 287]. We have shown that the upregulation of TWIST expression in gastric adenocarcinoma cells cultured in *Hp*-AGF secretome depended on EGFR detected at 96 h of experimental procedure and disappeared after the addition of EGFR inhibitor Tyrphostin A46. Tyrphostin also diminished the expression of *Snail*, *c-Myc*, *Oct4*, and *Sox-2* genes, which is consistent with the supportive role of EGFR in EMT, stemness, and GC development [13, 288, 289]. At the same time, it has been proposed that while the presence of TGF-β itself triggers EMT type 2, the parallel administration of TGF-β and HGF/c-Met is a potent EMT type 3 inducer leading to dynamic actin reorganization in epithelial cells [130, 289–292]. Accordingly, Kubiczkova and coworkers [293] have shown complementary activation of HGF receptor cMet in the tissues where TGF-βRII had been suppressed. HGF/c-Met pathway has been shown to be essential for growth, survival, and invasiveness of GC [294] with MMP2 and MMP9 being responsible for HGF release from the ECM and its processing to active form [295–298]. In turn, Spix and coworkers postulated that HGF promotes motility and tumor progression in part by EGFR activation which elevated level is frequently observed in GC; also, this is induced by *Hp* infection [299]. It has been stated that activation of c-Met participates in proteolytic cleavage of EGF ligand precursors located in the cell membrane, which can then activate EGFR (the triple membrane-passing signaling mechanism) [300, 301]. This interaction seems to be reciprocal, as increased c-Met protein level after increased EGFR activation has also been reported [302]. Moreover, it has been shown that TGF-β over-released by activated fibroblasts [302], including *Hp*-AGFs [10], can lead to production of several mitogenic growth factors including EGF [302]. Consequently, EGFR has been proposed to potentiate TGF-β induction of a subset of invasion-associated genes, along with transcriptional regulation of HBEGF, a heparin-binding EGFR ligand [302]. This phenomenon could give additional explanation of decreased expression of TGβRII active form in *Hp*-AGF secretome-reprogrammed epithelial cells, which on one hand allowed the escape from TGF-β-induced arrest of proliferation and on the other hand still sustained cell pro-pluripotent [12] and invasive phenotype [13]. The sustained activation of TGF-β elicited also by autologous TGF-β release [12], as well as increased expression of c-Met and EGFR [13], points to the role of reciprocal interactions between TGF-β and tyrosine kinase receptor pathways in reprogramming of epithelial cells [12, 13]. The alterations of TGF-β signaling occur in almost all GI tumors [137, 303] with TGF-β being either a tumor suppressor or a promoter depending on the temporal stage of the disease [137, 304]. In GC, SMAD3 is frequently downregulated [137, 305]. It has been proposed that TGF-β can antagonize BMP-like responses by the formation of pSMAD1/5–pSMAD3 complex which specifically bind to BRE suppressing transcription of downstream genes [137, 254]. Inactivation of SMAD3 in cancer diminishes the antagonism of TGF-β on BMP responses and enables TGF-β to induce BRE downstream tumor promoting Id genes [137, 233, 234, 254, 265] normally suppressed by canonical TGF-β signaling [137, 306, 307]. Additionally to reduce expression of BMP, GC cells are also characterized with reduced expression of activin membrane-bound inhibitor (BAMBI), an event also present in GC cells infected with *Hp*. BAMBI is a pseudo-receptor which negatively regulates TGF-β signaling by the inhibition of an active receptor complex formation to the level allowing EMT type 3 commitment [308], and it would be interesting to check if *Hp*-AGFs facilitate this process. Finally, TGF-βs mediate the migration of MSCs from bone marrow, peripheral blood, or surrounding tissue to be integrated into the injured/remodeling tissues. Then, TGF-β1 induces SMAD3-dependent nuclear accumulation of β-catenin, stimulating MSC proliferation. On the other hand, BMP2 antagonizes WNT3a signaling and inhibits proliferation of MSCs through interaction of SMAD1/5 with Dsh-1 [255, 309]. In addition, TGF-β1 induces differentiation of BM-MSCs into myofibroblasts and their expansion combined with SDF-1α induced recruitment into tumor bed [255, 310]. Thus, in an autocrine fashion, SDF-1α functions as pro-proliferative factor, while in a paracrine fashion, as the chemoattractant or the remaining myofibroblasts [31, 255, 310]. TGF-β1 is also able to stimulate EndMT [186–188, 255]. Consequently, TGF-β1 is perceived as the important part of reciprocal communication between CSCs and CAFs [137, 255, 262, 265, 282, 293, 311] and it has been proposed that CAFs from the scirrhous GC might contribute to the maintenance of CSC properties *via* the TGF-β/p-SMAD2 signaling [312].

### Notch signaling pathway

The Notch signals are transmitted between adjacent cells in the process termed lateral inhibition, which is responsible for asymmetric stem cell divisions during tissue development, repair, and homeostasis [313, 314]. In mammals, there are four Notch receptors (NOTCH 1–4) and five Notch ligands (Delta-like (DLL) 1, 3, 4; and JAG 1, 2), which are all transmembrane proteins [313, 315]. It has been shown that Notch regulation of gastric antral and corpus progenitor cell proliferation occurs *via* NOTCH1 and NOTCH2 receptors [313, 316, 317]. Notch signaling is initiated by binding of the ligand expressed on the signal-sending cell to the receptor on the signal-receiving cell. This event initiates proteolytic cleavage in the intracellular signaling fragment of the receptor by ɤ-secretase complex, which leads to the release of the signaling Notch intracellular domain (NICD) [313, 318] which enters the nucleus. In the nucleus, NICD binds with a DNA-binding transcription factor CSL (CBF1/RBP-Jκ in mammals), displaces the CSL repressors, and recruits transcriptional coactivators, including CBP/p300, Spt6 transcription elongation factor, and Mastermind (MAML), thus converting a transcriptional repressor complex into an activator complex [319]. This complex can then activate transcription of Notch target genes which include c Myc, cyclin-D1, p21, and genes of NFκB pathway as well as the components of its own signaling cascade, such as the Notch ligand [313, 319]. In addition, the recruitment of different Notch modifiers, such as NUMB or GSK3β, modulates the Notch cytoplasmic recycling and protein–protein interactions, thus influencing Notch-induced transcription [315]. Notch signaling communicates short-range signals which are also short-lived due to receptor destruction and rapid degradation of NICD [313, 316, 320]. The lineage tracing experiments have found active NOTCH receptor signaling in adult stem cells within the mice stomach [313, 315, 321] and Notch has been established as a crucial pathway responsible for the regulation of gastric stem cell proliferation and differentiation. Notch inhibition by ɤ-secretase inhibitor (GSI) treatment in adult mice showed reduced progenitor cell proliferation in corpus and antrum [313, 320, 322] and, importantly, reduced number of proliferating LGR5^+^ antral stem cells. Cell sorter–isolated LGR5^+^ stem cells derived from GSI-treated mice initiated fewer organoids, which confirmed that Notch was required to support antral stem cell function [313, 321]. In contrast, constitutive Notch activation in LGR5^+^ antral stem cells induced stem and progenitor cell proliferation. Furthermore, gastric organoids derived from Notch-activated adult corpus and antral stem cells were shown to grow faster [313, 316, 321]. After Notch inhibition, the increase in antral epithelial cell differentiation has been observed, which underlines the importance of Notch signaling in stem cell maintenance in corpus and antrum [317, 321]. Concomitantly, the NICD-induced tumors are highly proliferative and are largely composed of undifferentiated cells expressing progenitor cell markers excluding LGR5 [321]. Accordingly, it has been shown that constitutive NICD expression in parietal progenitor cells, adult SOX2^+^ stem/progenitor corpus cells, and LGR5^+^ antral stem cells promoted formation of hyperproliferative glands in the mouse corpus and led to hyperplastic polyp development in corpus or antrum [316, 318, 320, 323]. Consequently, Notch pathway dysregulation, particularly *via* up-regulation of ligands and/or receptors, has been observed in human GC [320, 324–327]. Importantly, correlations between high expression of NOTCH1, NOTCH2, and JAG1 have been associated with GC morbidity [320, 324, 325]. NOTCH1 activation in human GC has been also associated with DLL1 promoter methylation [320, 326, 327] suggesting that epigenetic dysregulation of Notch signaling may also contribute to GC [321]. As summarized by Yao et al. [328], the crucial function of Notch signaling in GC is to stimulate CSCs; thus, Notch is consequently involved in EMT, radiotherapy endurance, and chemotherapy resistance with crucial role of autocrine and paracrine signaling between different compartments of tumor. Recently, it has been reported that NOTCH1 maintained a cancer stem cell–like phenotype in diffuse type GC by upregulation of a stem cell marker CD133 [326, 328]. It has been also shown that CAFs may facilitate migration and invasion of tumor cells through the Notch pathway [329]. Moreover, CAF-derived extracellular vesicles (EVs) were shown to contain transposable RNAs which activated STAT1 in cancer cells through the cytosolic pattern recognition receptor RIG-I. Subsequently activated STAT1 has been found to cooperate with juxtacrine-activated NOTCH3 to mediate Notch target gene transcription that supports resistance to chemotherapy and radiation [329, 330]. Therefore, it would be interesting to check EV release in *Hp*-AGFs. The cooperation between Notch and other signaling pathways has been also postulated. Zavadi showed that TGF-β led to EMT by induction of the expression of the hairy/enhancer-of-split-related transcriptional repressor (Hey1) and JAG1 [277]. Additionally, the interactions between Notch, STAT3, and TWIST were shown to promote development of GC. Notch1 enhanced both TWIST expression and phosphorylation of STAT3 in gastric adenocarcinoma SC-M1, HEK293, and K562 cells [325]. Moreover, the overexpression of NICD increased growth, metastasis, migration, and invasion of GC cells by enhancement of the interactions between STAT3 and Twist promoter [327, 328, 331]. Consequently, CAF-derived IL-6, which increased level is observed in *Hp*-AGFs parallelly to TWIST activation in *Hp*-AGF—reprogrammed gastric epithelial and adenocarcinoma cells [13], promoted stem cell–like properties in HCC cells by activating Notch signaling through the phosphorylation of STAT3 on Tyr705 [332]. Furthermore, Dll1^+^ breast cancer cells were shown to recruit CAFs in an IL-6-dependent fashion and to promote Wnt ligand secretion by NOTCH2/3-expressing CAFs resulting in Wnt/β-catenin–dependent increase in Dll1^+^CSCs to promote metastasis. The pharmacologic blockade of IL-6 or Dll1 suppressed CAF-dependent enhancement of Dll1^+^CSC function as well as metastasis in radioresistant tumors [333]. On the other hand, Notch has been found to drive the Notch-ON/Wnt-OFF state. At the membrane, independently of ligands, NOTCH1 can antagonize β-catenin by the endocytic mechanism sequestering β-catenin into the membrane fraction. Within the nucleus, the intracellular domain of Notch1 can also limit β-catenin-induced transcription [334]. Conversely, the genes encoding both Jag1 and an inhibitor of Notch signaling Numb are the targets of canonical Wnt/β-catenin signaling [335, 336]. Additionally, GSK3β mediating β-catenin degradation can phosphorylate NICD increasing its transcriptional activity and stability [337, 338]. It has been also assumed that CAF-derived HGF stimulation activates Notch. HGF activated Notch pathway by the upregulation of its ligands JAG1 and DLL4196, together with uPA in prostate cancer DU145 cells [339]. Parallelly, NOTCH1 positively regulated c-MET expression in human breast adenocarcinoma MDA-MB231 and HCC1143 cells [340]. Activation of the uPA system has been implicated as a rate-limiting step for tumor cell migration/invasion, responsible for the conversion of latent TGF-β to mature TGF-β [31]. UPA is also documented in the process of HGF activation [340, 341]. Above findings reflect reciprocal HGF/TGF-β interactions in *Hp*-AGF-induced propluripotent and proinvasive cancerogenic reprogramming of gastric epithelial and adenocarcinoma cells [12, 13].

### Non-coding RNAs

microRNAs are small, highly conserved non-coding RNA molecules involved in the regulation of gene expression through the binding of the target mRNA to inhibit transcription. For example, members of the miR-200 family: miR-141, -200a, 200b, 200c and -429, were shown to target different signaling pathways including the Notch, Wnt, and TGF-β pathways, thus influencing events like cell renewal, EMT, and cell migration [342]. For instance, members of the miR-200 family target the 3′ untranslated regions of JAG1 to adjust its level in human tissues [343]. The miR-200 family has been identified to be crucial in tumorigenesis, and consequently, dysregulation of miRNA expression profiles has been demonstrated in most examined tumors [344–346]. In the metastatic cascade, miR-200 exerts a context-dependent role. In general, the expression of miR-200 family members in the primary tumor strengthens an epithelial phenotype, thereby preventing EMT; nevertheless, miR-200s also support MET at distant organ sites, thereby promoting metastatic colonization [183]. miR-141 was shown to directly target and repress TGF-β2, thus promoting epithelial phenotype in TGF-βRII-expressive cell line [347, 348], whereas TGF-β1 evoked transcriptional repression of *miRNA-200* family genes through the regulation of histone H3 methylation [347, 349]. Additionally, it has been found that increased proliferation of TGF-βRII-deficient cell lines was associated with increased expression of miR-200b [347, 348]. Accordingly, it would be interesting to correlate enhanced proliferation of *Hp*-AGF reprogrammed epithelial cells characterized by decreased level of active, glycosylated form of TGF-βRII [12] with miR-200 protein expression. O’Brien and coworkers have underlined that clinical studies in CRC patients have shown decreased primary tumor tissue expression of miR-429, miR-200a, and miR-200c, while the serum levels of miR-141, miR-200a, and miR-200c were increased which may be mediated by differential TGF-βRII expression [347]. Izmuchenko reported that the miR-200 family regulating the expression of factors implicated in tumor metastasis, e.g., E-cadherin and ZEB1 [350], is significantly downregulated in the triple-negative breast cancer cells undergoing EMT in response to TGF-β. He also showed that during TGFβ-mediated EMT, inhibition of the miR200 family results in upregulated expression of the mitogen-inducible gene 6 (MIG6), a negative regulator of EGFR, which leads to the EMT-associated AKT-activated EGFR-independent state that induces resistance to EGFR inhibitors [350]. Shaalan and coworkers investigated long non-coding RNA (LncRNA) H19 and related miRNAs including miR-139 and miR-200 in the plasma samples of treatment responsive gastric ulcer (GU) *vs* nonresponsive GC patients**.** All recruited patients were diagnosed with *Hp* infection. They found that downregulated serum miRNA 200c/miRNA 139 expression levels could provide a new potential prognostic panel for GU predictive response and together with LncRNA H19 could serve as a potential diagnostic biomarker for early GC diagnosis [351]. Other reports have depicted the regulatory role of miRNAs in *Hp*-induced inflammatory response characterized by upregulated miR-146a and strong miR-155 induction. It has been shown that MiR-155 correlated with early release of proinflammatory TNF-α and IL-6 [350, 352]. Consequently, NFκB which is activated during *Hp*-induced infection has been reported to act as the transactivator of miR-200b and miR-200c [353]. In colorectal cancer, CAFs were shown to upregulate long noncoding urothelial carcinoma-associated 1 RNA (lncRNA UCA1) in cancer cells leading to upregulation of mTOR. Upregulation of UCA1/mTOR axis suppressed p27 and miR-143 leading to an increase in the expression of cyclin-D1 and KRAS and thus EMT combined with intensification of migration, invasion, and cell proliferation [354]. It has also been shown that several miRNAs were regulated by hypoxia. For example, miR-210 involved in several hypoxia-related cancer cell signaling pathways was upregulated by the HIF1-α transcription factor. Also, miR-34 and miR-199a were significantly downregulated under hypoxic conditions, leading to EMT and associated stem cell phenotypes [355]. Cells under hypoxic conditions showed consistent upregulation of the set of pro-metastatic genes [355–357]. In colorectal cancer, downregulation of miR-34 which targets *NOTCH1* and *JAG1* involved in cancer stem cell acquisition resulted in increased IL-6 signaling, which led to EMT and cancer metastasis [358]. Importantly, CAF-derived exosomes are established as potent source of miRNAs [359, 360]. CAFs can enhance invasion and migration in GC through the miR-106b/ phosphatase and tensin homolog (PTEN) pathway [361]. Also, it has been demonstrated that GC-originated mesenchymal stem cells facilitated GC progression and development through the transfer of exosomal miR-221 to GC cells [362, 363]. It has been also shown that downregulated CAF-derived miRNAs can also contribute to the migration of tumor cells [363, 364]. In GC, miR-214 and miR-139 are significantly downregulated and CAF-derived low-expressed miR-214 removes the inhibition of EMT, inducing E- to N-cadherin switch resulting in enhanced migration and invasion of GC [364, 365]. Moreover, GC cell line cocultured with GC fibroblasts (GCFs) led to the reduction of miRNA-34 expression in all GC cell lines compared with untreated cells. Importantly, among miRNA-34 targets one can distinguish CDK4, CDK6, c-MET, E2F3, E2F5, and N-Myc. Reciprocally, the level of miRNA-34 was also decreased in GCFs in response to coculture with GC cell lines. Collectively, these results demonstrated that miRNA-34 was downregulated in GC cells and neighboring GCFs [360]. The up-regulated CAF-derived miRNAs are associated with a positive feedback loop for tumor progression [366]. For example, CAF-exo showed higher expression of miR-17-5p which subsequently influenced CRC metastatic capacity and directly targeted 3′-untranslated regions (UTRs) of RUNX family transcription factor 3 (RUNX3) which then interacted with protooncogene MYC. Both RUNX3 and MYC bound to the promoter of TGF-β1 activating the TGF-β signaling pathway and contributing to tumor progression. In addition, RUNX3/MYC/TGF-β1 signaling sustained autocrine TGF-β1 to activate CAFs, and activated CAFs released more exosomal miR-17-5p to CRC cells, forming a positive feedback loop for CRC progression [366, 367]. It has been also shown that dysregulation of CAF-derived miRNAs mediates the chemoresistance of CAFs and tumor cells to chemotherapeutics. For instance, in CRC, upregulated CAF-derived miR-93-5p is responsible for the radioresistance of cancer cells by targeting FOXA1 through the activation of the TGF-β pathway [368].

## *Hp* virulence factors and host genetic polymorphisms in the risk of GC development

5.5% of all cancers and more than 60% of GC cases are caused by *Hp* infection [369, 370]. However, although between 40 and 80% people are infected with *Hp*, only around 3% is reported to develop GC [371, 372]. It has been postulated that *Hp* has evolved during long cohabitation with humans, and it seems that the co-evolutionary relationships are the main determining risk factors for gastric disease [369, 373]. The risk of cancer due to *Hp* infection varies in the different geographical regions of the world which can be partly attributed to the different *Hp* genotypes circulating in various geographic regions [369, 374]. *Hp* exhibits genetic diversity across the species [371, 375, 376], nucleotide sequence diversity of the individual genes associated with the high rates of mutation, and high level of recombination across the species [335, 371, 377]. To date, seven bacterial population types have been observed, hpAfrica1 (hspSAfrica, hspWAfrica, and hspCAfrica), hpAfrica2, hpNEAfrica, hpEurope, hpSahul, hpAsia2, and hpEastAsia (hspEAsia, hspAmerind, and hspMaori), which differ in their geographical region of appearance and differ in their ability to evoke GC, premalignant histologic lesions, and DNA damage. Thus, the strains from different human hosts are distinct, including the sequences of the specific genes, variations in the contents of the genes, and the organization of chromosomes [336, 369, 371, 378, 379]. Also, the gastric section of the host is comprised of low diversity of bacteria, while it is rich in terms of the genetic variants among the distinct subpopulations of the *Hp* which allows the bacterium to resist protective mechanisms present in the individual hosts. The primary genome of *Hp* is comprised of 1100 genes that are exhibited in all strains, with each of the strains comprising of other hundreds of additional genes [369, 371, 380]. The adhesion of *Hp* to glycan epitopes on the cell surface is a crucial step for a successful colonization*. Hp* OMPs are crucial for adaptation of the pathogen to the host. Genes encoding outer membrane proteins (OMPs) consist of 4% of *Hp* genome and include *Hp* outer membrane proteins (Hop) and Hop-related proteins (Hor), e.g., BabA (HopS), SabA (HopP), and HorB [369, 371, 380]. The most extensively researched outer membranes BabA and SabA were shown to correlate with the progression of inflammation of the gastric gland and high risks of GC development [369, 371, 380, 381]. As not every *Hp* strain expresses functional BabA or SabA adhesins, other bacterial proteins are most probably also involved in the adhesion process. The alpAB locus has been shown to encode the outer membrane adhesins AlpA and AlpB, which bind laminin [380, 382]. Despite the multiple adhesins employed by *Hp* to attach to gastric epithelial cells, the deletion of *AlpAB* alone reduced bacterial binding by 60–70%, and reduced *Hp* colonization. The HorB protein is another adhesin with so far unidentified ligand [380, 383]. HopH, encoded by the *HP0638/hopH* gene, belongs to *Hp* virulence markers including *vacAs1*, *vacAm1*, *babA2*, and particularly *cagA* [380, 384]. The association of the *hopH* gene with gastric disorders could be due to promotion of increased bacterial adherence and colonization mediated by the hopH. The expression of hopH has been found to be regulated by phase variation within a CT dinucleotide repeat motif [380, 384]. The *cagA* and *vacA* genes are the two main determinants of *Hp*-associated disease risk that are mainly involved in the chronic gastritis and damage of epithelial cells leading to GC [369, 371, 380, 385]. *VacA* gene signal (*s*), intermediate (*i*), and middle (*m*) regions include the *s1* (*s1a*, *s1b*, and *s1c*), *s2*, *i1*, *i2*, *m1*, and *m2* subtypes [369, 386]. Region *i* plays an important role in the vacuole-creating activity linked with GC [369, 380, 387]. This relationship is independent of and much greater in magnitude than the associations of the *s*- or *m*-types of *vacA* and *cagA* with GC [369, 388]. VacA virulence depends on the combination of individual genotypes. The *vacA s1/m1* alleles determine high cytotoxic activity of VacA, whereas the *s1/m2* and *s2/m2* genotypes are not cytotoxic. The s1/m1 profile is strongly correlated with the outcome of duodenal ulcers, peptic ulcer disease, progression of preneoplastic lesions, and formation of GC [369, 380, 387, 389].

Recently, the deletion of 81 bp (*d* region: *d1* or *d2*) between the *m* and i regions has been reported. Without such deletion, it is classified as *d1* or *d2* if a 69 to 89 base pair deletion is present [380, 389, 390]. It was found that *i1* variants of the VacA protein have stronger vacuolating activity than *i2* variants, thereby is considered a better predictor of disease severity than the *s1* and *m1* variants in Western strains. The *i* region may contain A, B, and C polymorphic domains with VacA toxicity depending on B and C part [380, 387]. The *vacA d1* genotype shows a strong association with the neutrophil infiltration and gastric mucosal atrophy in both the corpus and the antrum [369, 381]. The highest frequencies of the pathogenic *vacA* alleles (except *m1*) and the cagA+ genotype are found in the areas with high incidence of GC, although there is often no association between these genotypes and the risk of GC [369, 371, 380]. *Hp* strains with the *vacA s1*, *i1*, *m1*, or *d1* genotypes from Western countries are potentially linked with an increased neutrophil infiltration and gastric mucosal atrophy [380] and strongly associated with the risk of GC. In general, independent of the *Hp* population types, bacterial strains with the *vacA s1m1i1d1c1* genotype exhibited greater inflammatory responses and atrophy in the antrum, unlike strains with the *vacA s2m2i2d2c2*, *s2m2i2d2c2*, or *s1m2i1d1c2* genotypes [369]. Recently, a novel five- and six-genotype combinations (*vacA s1m1i1d1c1*, *s1m2i1d2c1*, *s1m2i2d2c1*, and *s1m2i2d2c1cagA*) have been linked with the risk of GC. These associations were mainly dependent on the presence of *c1* type of *vacA*. Therefore, according to Bakhti and co-workers, analysis of all the combined genotypes of the *vacA* alleles and the *cagA* status may play a critical role in predicting clinical outcomes associated with *Hp* [369, 391]. Cross-sectional study in China showed that individuals seropositive to Omp, CagA, VacA, and HP0305 significantly increased the risk of precancerous lesions and the risk estimate was increased by seropositivity to 4, 5, or more than 6 specific *Hp* antigens (Omp, HP0305, HyuA, HpaA, CagA, and VacA) relative to seropositivity to 3 or less than 3 antigens. However, the risk was very high when the individuals were seropositive to both Omp and HP0305. This strongly suggests that in East Asia these serological biomarkers are more important than CagA seropositivity alone for the risk of advanced gastric lesions [369, 392].

CagA protein from various *Hp* strains presents a wide diversity at the carboxyl terminus that includes repetitive phosphorylation sites (EPIYA motif) [369, 393]. EPIYA motifs determine CagA interaction with numerous host proteins influencing cytoskeleton, cell-cell adhesions, and a variety of gene being expressed [369, 371, 380, 393]. The diversity of EPIYA motif can influence the CagA structure and its interaction inside the cells [369, 371, 380, 394]. Based on the sequences surrounding the EPIYA motif, EPIYA-A, EPIYA-B, EPIYA-C, or EPIYA-D segments have been specified [369, 380] and their combination may vary depending on geographic regions [369, 380, 395]. In general, Western *Hp* strains possess EPIYA -A, -B, and -C whereas strains from East Asian region EPIYA -A, -B, and -D. The East Asian CagA-positive *Hp* strains are more closely associated with GC [369, 380]. Phosphorylated CagA regions are primarily EPIYA-C and -D sites, which are required for binding to SHP-2 and its activation [369, 380, 395, 396]. The association between the number of EPIYA-C regions and increased CagA tyrosine phosphorylation SHP-2 binding activity, cytoskeletal alterations, IL-8 expression in gastric mucosa, development of the hummingbird cell phenotype, and severe disease frequency has been reported [380, 397]. Regardless of the C/D type, most CagA molecules include single A- and B- tyrosine phosphorylation motifs (TPMs). Phosphorylated A- or B-TPMs have host interaction partners distinct from C- or D-TPMs, suggesting unique signaling functions. Zhang showed that in the Western population, the polymorphism of the EPIYA-B motifs can influence the frequency of disease development [380, 398].

In contrast, the recent study conducted in East China showed that EPIYA-ABD had no relation with gastric diseases (chronic gastritis and gastric or duodenal ulcerations), and only polymorphism at amino-acids 878 and 879, flanking the EPIYA-A motif, had a significant association with GC [369, 399]. In addition, other EPIYA-like motifs have been identified (EPIYT, ESIYT, ESIYA, GSIYD). The analysis carried out by Zhang and coworkers demonstrated that the association of EPIYT segments with GC is lower than the EPIYA motifs [380, 398].

Infection with *Hp* is likely to spread throughout a family, which is partially related to intrafamilial transmission of *Hp*. Nevertheless, based on the familial clustering on GC [163, 400], genetic susceptibility predisposing to more intense immune response to *Hp* and consequently to GC development has been introduced [400, 401]. The various groups of host receptors are simultaneously engaged in recognition of *Hp* compounds and the development of GC, e.g., TLRs, nucleotide-binding oligomerization domain (NOD)-like receptors (NLRs), dendritic cell–specific intercellular grabbing non-integrin; retinoic acid–inducible gene (RIG)-I-like receptors (RIG-I); and melanoma differentiation–associated protein 5. Polymorphisms in genes, which are involved in the signaling cascades *via* TLR, NLR, apoptosis-associated speck-like protein, and caspase recruitment domain containing protein 8 (CARD8), can modulate the host immune response during *Hp* infection [114, 369, 371, 380, 400] and increase the risk of GC [114, 380, 400, 402]. As mentioned earlier, the activation of TLR-4 leads to MAPK pathway activation, which results in the activation of several transcription factors eliciting altered differentiation, uncontrolled growth, and proliferation leading to GC development [400, 403]. It also leads to activation of the proinflammatory NF-κB pathway [400, 404]. TLR2 signaling is also involved in the expression of the nuclear factor NF-κB and both TLR4 and TLR2 are engaged in the response of host immune cells against *Hp*, which effectiveness depends on the polymorphism of those receptors [114, 380]. Accordingly, much attention has been paid to the influence of *TLR receptor* polymorphisms on the development of *Hp*-induced GC. It has been found that single-nucleotide polymorphisms (SNPs) of the *TLR4 receptor* were connected with an increased risk, including *TLR4 rs4986790* (Asp299Gly), *TLR4 rs4986791* (Thr399Ile), *TLR4 rs10116253*, *TLR4 rs10983755*, *TLR4 rs11536889 (C3725G/C*), and *TLR4 rs1927911*. Among many polymorphisms, the TLR4 Asp299Gly and Thr399Ile polymorphisms have been considered the most important [114, 380, 405, 406]. Meta-analysis of *TLR2*-196 to -174 deletion and risk of GC conducted on 1364 GC patients showed that there is an association between this polymorphism and risk of GC in the Japanese population. Polymorphism at this position decreases the induction of IL-8 secretion, thus impairing the response to *Hp*. During *Hp* infection, monocytes and macrophages have been shown to release IL-12 in response to CD14-dependent activation which was correlated with the infiltration of gastric mucosa with T helper 1 lymphocytes and chronic inflammatory response [380, 407]. Two SNPs identified in the promoter region of the *CD14* gene: *-260C/T* (*rs2569190* or *CD14 -159*) and -561C/T (*rs5744455*), have been suggested to increase the susceptibility to GC [47, 48, 380]. The *CD14-260* T allele had decreased affinity for the binding with DNA of transcription factors such as stimulatory proteins (SP) 1, SP2, and SP3 of which SP3 downregulates the activation of the cells by SP1 and SP2. Thus, the SP3 to SP1 and SP2 ratio is considered to play an important role in the regulation of CD14 transcription [380, 408, 409].

It has been observed that patients who progress to atrophy and cancer secrete lower levels of gastric acid compared with patients with duodenal ulcers [380, 410]. Thus, the initial genetic study of families with an increased incidence of precancerous changes focused on IL-1β, a well-known inhibitor of acid secretion in the stomach and proinflammatory cytokine. The *IL-1β* gene cluster includes *IL-1β* and *IL-1RN*, the gene that encodes IL-1 receptor antagonist. The initiation or the maintenance of inflammation depends on the balance between IL-1β and IL-1Ra [380, 410]. The most intensively studied *IL-1RN* polymorphism connected to GC outcome is a 86-bp variable number of tandem repeat polymorphism in the *IL-1RN* second intron (IL-1RN*2) [380, 411]. The study carried out on the Brazilian Amazon population by Melo Barbosa and coworkers showed that among patients with gastric ulcer and adenocarcinoma there was a higher frequency of allele 2 carriers (IL-1RN*2). The presence of the IL-1RN*2 variant served as explanatory for the increased levels of IL-1β in the gastric mucosa and hypochlorhydria, events not observed in IL-1RN1/1 variant [380, 412].

Also, TNF-α activity and concentration can be influenced by SNPs (G to A transitions at -308A and -238 positions) in the promoter region of *TNF-α gene* [380, 413, 414]. Sun and coworkers [56, 380] demonstrated that *TNF-α*-308G/A and -1031 T/C polymorphisms may be protective factors against *Hp* infection, whereas -863C/A substitution may be a risk factor, particularly in Asian populations. IL-10 is a pleiotropic cytokine, which can suppress or stimulate anti-cancer properties of immune cells. IL10 downregulates the production of pro-inflammatory cytokines inhibiting Th1 lymphocytes, while stimulating B and Th2 lymphocytes [380, 415]. The association between *IL-10-592 A/C* SNP and susceptibility to GC has been postulated [380, 416]. Moreover, IL-8 polymorphism leads to elevated production of IL-8 and intensified inflammatory response. Ohyauchi and coworkers showed that in *Hp*-infected patients, the presence of *IL-8-251A* allele was linked with the gastric ulcer, gastric atrophy, and then cancer [380, 417]. In addition to cytokine polymorphisms, genetic differentiation of COX also plays an important role in the development of *Hp*-associated gastric diseases [380, 418, 419]. Concerning the polymorphisms of promoter region of *COX-2* (*1195G/A* and *-765G/C*), Li and coworkers [418] showed that the increased risk of GC appears in the carriers of the *COX-2*-*1195AA* but not of the *COX2-765G/C* genotype. Meta-analysis conducted by Zhao showed that the *-765G/C* polymorphism (*rs20417*) in the promoter region of the *COX-2* gene could be a risk factor for GC in Asians and Indians. The *COX-2-765G/C* polymorphism was significantly associated with an increased risk of GC, regardless of *Hp* infection [419].

Assuming, as Bakhti brilliantly underlined, it seems that the main determining risk factors for gastric diseases are the co-evolutionary relationships between host and bacteria. This interaction may include the ethnic composition of hosts including the receptors engaged in recognition of *Hp* compounds, pro-inflammatory cytokine polymorphisms, and other factors playing an important role in the *Hp* colonization of incoming strains, differences in transmission ecology, influencing the composition of *Hp* populations [369].

Due to development of the next-generation sequencing (NGS), the genetic studies of complex phenotypes are shifting to include the lower frequencies of rare variants, which is based on “common disease–rare variant” hypothesis. According to this hypothesis, the multiple rare variants which represent the larger effect sizes are the important determinants of disease development and heritability [369]. The participation of fibroblast *Hp* infection in cancer development seems to constitute an important component of this process, but the research on the diversification of fibroblast versus epithelial and immune cell infection and their activation is still missing. Most importantly, the question of what exactly happens if the *Hp* infection develops mainly within connective tissue is so far unresolved, as the focus has been addressed to *Hp*/epithelial interactions. In the light of the recent studies showing GC development in initially asymptomatic patients [15] and participation of *Hp* in GIST development [14], the *Hp*-induced fibroblast activation may participate in the gradual development of these disorders. To properly address the pathological series of events, the genotypic composition of the bacteria allowing it to penetrate mucous and epithelial barrier, further adherence, and destabilization of the crucial signaling pathways responsible for disturbed fibroblast/stem cell communication should be taken into account together with an impaired immunological response of the host.

## Conclusions

Despite considerable improvement in the early diagnosing and anti-cancer therapies, GC remains the fourth most common cause of cancer-related death in the world with 90% of all stomach tumors being malignant. GC is heterogeneous and contains cells with various stem cell properties referred to as CSCs. Recent advances in the field provided evidence that gastric CSCs are likely the result from dedifferentiation of metaplastic lineages including spasmolytic polypeptide expressing metaplasia (SPEM) and intestinal metaplasia (IM), normal adult gastric progenitor cells (GPCs)/stem cells, or bone marrow–derived mesenchymal stem cells (BM-MSCs). This process of dedifferentiation, recruitment, and transformation occurs due to the changes in the gene expression due to the influence of external environment, e.g., the continuous action of inflammatory mediators and stimulation by carcinogens like *Hp*.

Recently, the correlation between asymptomatic *Hp* infections and subsequent GC development has been reported underlying the importance of reciprocal interaction between microenvironment and stem cell compartment. These interactions employ numerous signaling pathways including Wnt, Notch, TGF-β/BMP, and non-coding RNAs. The changes introduced to any component of stem cell/niche system may irreversibly disturb this sophisticated system for information exchange and thus interfere the proliferation/differentiation balance, leading to neoplastic transformation. One of the main components of the stem cell/niche system are fibroblasts and myofibroblasts. We have previously reported that *Hp* (*cagA+ vacA+*) increases the number of myofibroblast through the FMyoT, EMT, MSC, and BM-MSC differentiation and possibly EndMT and can further directly or indirectly activate them towards cells possessing the properties of CAFs. In contrast to normal fibroblasts and myofibroblasts, CAFs become constantly activated and neither revert to a normal phenotype nor undergo apoptosis and elimination. They contribute to cancer-related inflammation, tumor cell proliferation, EMT type 3, invasion, and metastasis as well as chemo- and immunoresistance. This process involves such STAT3/NFκB pathway targets as TGF-β1, HGF, osteopontin (OPN), IL-1β, IL-6, IL-8, SDF-1, or HIF-1α next to glucose metabolism reprogramming and ROS induction eliciting further reprogramming of mesenchymal and epithelial progenitor/stem cells. Other downstream targets of STAT3/NFκB interactions: SNAIL, ZEB, and TWIST expression, seem to provide *Hp*-AGFs with additional properties like CAF-specific secretome. Thus, the targeting NFκB/STAT3-dependent EMT-TFs—particularly TWIST activation—offer a great promise as one of the possible targets in cancer pharmacotherapy. The *Hp*-activated fibroblasts may further participate in cancerogenic expansion of stem and progenitor cells through the changes in main signaling pathways involved in the proper balance between proliferation and differentiation within stem cell compartment. *Hp* infection elicits deregulated expression of Wnt ligands and signaling regulators, decreased level of BMP ligands, and overexpression of BMP signaling inhibitors. It also leads to oversecretion and increased activation of TGF-β1 resulting in the recruitment of excessive progenitors, or trans-differentiation/reprogramming of resident cells through the direct activation of target genes and multiple downstream signaling pathways like Ras/Raf/MEK/ERK/MAPK or p38 and JNK. The SMAD-independent, non-canonical TGF-β pathways are considered important effectors for tyrosine kinase receptors. TGF-β and other *Hp*-AGF over-released growth factors and cytokines may also increase Notch signaling, e.g., through the induction of Notch receptor and their ligands, including JAG1, which is an important part of molecular machinery regulating cell fate decisions. All these downstream pathways interact with one another often operating in a positive feedback loop and are additionally influenced by the presence of gene expression modulators, as non-coding RNAs.

Assuming the basic regulatory role performed by fibroblasts, we believe that *Hp*-induced fibroblast activation is an important component of positive feedback loop involved in chronic inflammation engaging multiple cell types with inseparable reciprocal interactions between these cells, which may ultimately lead to cancer development. The *Hp*-activated fibroblasts influence not only stem cell and progenitor zone of gastric glands, but also greatly influence epithelial and immune cells; however, in the light of the identification of the immune-modulating population iCAF, myCAF phenotype [84], and additional functional subsets that resemble both the iCAF phenotype and the myCAF phenotype, future studies on *Hp* influence should focus on precise identification of the activated fibroblast phenotype/phenotypes. It was reported that iCAF clusters were associated with CD8+ T cell infiltration and a positive response to immunotherapy, whereas myCAFs were associated with resistance to therapy [84, 420]. This association may be mediated by the ability of myCAFs to promote regulatory T cell differentiation and upregulation of immune checkpoint molecules. The presence of this discrete pathogenic population with pro-inflammatory fibroblasts expressing cytokines and chemokines, contributing to inflammatory reaction development and maintenance, while a separate subset produces and activates connective tissue components and remodeling enzymes leading to fibrosis [84, 420, 421], may cooperate in chronic inflammatory diseases and cancer development, e.g., in *linitis plastica* [84, 422]. The exact CAF phenotype is also important due to the possible polarization of TAMs, which are the most numerous immune cells in the tumor microenvironment. TAMs are divided into two subtypes: pro-inflammatory M1 and immunosuppressive, conditioned by the tumor microenvironment M2 [423, 424]. Growth factors, cytokines, and chemokines, including TGF-β, PGE2, IL-6, and CCL5 which are also released from *Hp*-AGR, potentially modulate the polarization of monocytes mainly towards M2 macrophages that not only provide immunosuppression, but further contribute to promotion of tissue remodeling and angiogenesis, additionally secreting cytokines and growth factors that in turn promote both tumor growth and progression of the tumor, and reciprocally influence fibroblasts and stem/progenitor cells [423–425]. Additionally, Biffi and co-workers demonstrated plasticity between the iCAF and myCAF states, which are regulated by the surrounding microenvironment, e.g., by the presence of TGFβ, TNF, and PU.1 [84, 426].

Moreover, the activation of fibroblasts generally occurs by similar signaling networks with next to matrix stiffness, the key role of common NF-κB and JAK/STAT pathways. Activation of these pathways is induced by a variety of signals, including TLRs, TNF, IL-6, IL-1β, or TGFβ [84, 426, 427]; thus, the activation may be triggered directly by *Hp* or may come from infected and transformed epithelial cells, immune cells, and other directly or indirectly activated fibroblasts with again further positive feedbacks. In the light of above conclusions, *Hp*-activated fibroblasts, especially by highly procarcinogenic *Hp* strains, seem to constitute the target for anti-cancer therapies and importantly for the pharmacotherapy diminishing fibroblast activation particularly at the early stages of *Hp* infection.
